# Stage 2 Registered Report: Refinement of tickling protocols to improve positive animal welfare in laboratory rats

**DOI:** 10.12688/f1000research.166417.2

**Published:** 2025-09-23

**Authors:** Vincent Bombail, Sarah M Brown, Cammy Beyts, Jessica E Martin, Tianhao Li, Simone L Meddle, Michael Mendl, Emma SJ Robinson, Tayla J Hammond, Birte L Nielsen, Megan R LaFollette, Ignacio Vinuela-Fernandez, Emma KL Tivey, Alistair B Lawrence

**Affiliations:** 1 Animal Behaviour and Welfare group, Scotland's Rural College, Edinburgh, Scotland, UK; 2 The Roslin Institute and Royal (Dick) School of Veterinary Studies, The University of Edinburgh, Roslin, Scotland, UK; 3 Bristol Veterinary School, University of Bristol, Langford House, Langford, BS40 5DU, UK; 4 Biomedical Sciences Building, University Walk, University of Bristol, School of Physiology, Pharmacology and Neuroscience, Bristol, BS8 1TD, UK; 5 Universities Federation for Animal Welfare, Wheathampstead, England, UK; 6 The North American 3Rs Collaborative, Denver, CO 80202, USA; 7 Bioresearch Veterinary Services, University of Edinburgh, Edinburgh, EH16 4SB, UK

**Keywords:** Rat tickling; Playful handling; Pinning; 50 kHz USVs; Sex differences; Individual variation

## Abstract

**Background:**

Rat tickling (heterospecific play between rats and the human hand), can promote positive affect and improve laboratory rat welfare. However, individual variation, particularly in response to the standard tickling protocol involving frequent pinning, may limit its effectiveness. Following hypothesis registration ( https://f1000research.com/articles/11-1053/v2), we aimed to refine the protocol by testing responses to different amounts of pinning during playful handling (PH), a flexible form of tactile interaction resembling juvenile rough and tumble rat play.

**Methods:**

Juvenile male and female Wistar rats received six daily 30-second sessions of PH with 0, 1, or 4 pins per session. A non-PH control involved a motionless hand. The primary response variables were the count and variability of 50 kHz ultrasonic vocalisations (USVs), which are measures of positive affect in rats. Additional exploratory outcomes included behavioural and physiological responses in the elevated plus maze and open field tests.

**Results:**

All PH treatments increased 50 kHz USVs compared to controls, confirming their positive effect. Males showed no difference in USVs across pinning conditions, while females vocalised more in treatments with minimal or no pinning. Except for the highest pinning treatment (P4), females produced more USVs than males across all conditions, including controls. However, reducing pinning did not reliably decrease USV variability or alter behavioural and physiological outcomes.

**Conclusions:**

Applying 50 kHz USVs as a measure of positive affect, these results confirm that tickling induces positive affect in juvenile laboratory rats. We report the novel finding of sex differences in response to tickling. Females preferred tickling where the amount of pinning is minimised relative to males that showed no preference across tickling treatments. Levels of USVs suggest that females possibly found the applied treatments, including control conditions, more rewarding than males did

Research highlights **Scientific benefits:**

• A scientifically based refinement of rat tickling protocols with treatments varying in amount of pinning applied to both sexes.
• 50 kHz ultrasonic vocalisations (USVs) used as a measure of positive affect and shown to be responsive to treatments.
• Demonstration of the benefits of considering sex differences where the objective is to improve welfare with female juvenile Wistar rats preferring tickling with less pinning relative to males. We were not able to confirm our hypothesis that tickling with less pinning reduces the variability of response to tickling.

**3Rs benefits:**

• Tickling protocols should be adjusted:
  ○ Female rats prefer tickling with 1 pin per session;
  ○ In males the 1 pin per session treatment was the numerically highest treatment in terms of USVs;
  ○ Harmonising to one pin per session for both sexes would be consistent with our findings and help with implementation across facilities.
• Rat tickling has positive effects on rat affective state; 50 kHz calls increased after one 30-second session.
• Sex differences should be addressed when developing welfare interventions.

**Practical benefits:**

• 50 kHz USVs can be used to refine welfare interventions reflecting rats’ affective state.
• This paper provides the basis of a refined tickling protocol that can be used for both sexes.

**Current applications:**

• This study used juvenile Wistar rats of both sexes aged between 3-4 weeks and 7 weeks. Rats were paired housed in same sex groups and tested in a separate arena after habituation. Separate groups (n = 16 in each group) were exposed to control (stationary hand present) and tickling treatments with one 30 second session/day over 6 days (P0 – rats playfully handled but not pinned; P1 – rats playfully handled with 1 pin/ session; P4 – rats playfully handled with 4 pins/session).

**Potential applications:**

• Results should be extended to older animals and other strains.
• Further validation of our findings using behavioural tests of affective state.

## 1. Introduction

### 1.1 Research question: background, importance and relevance

**Figure 1.**
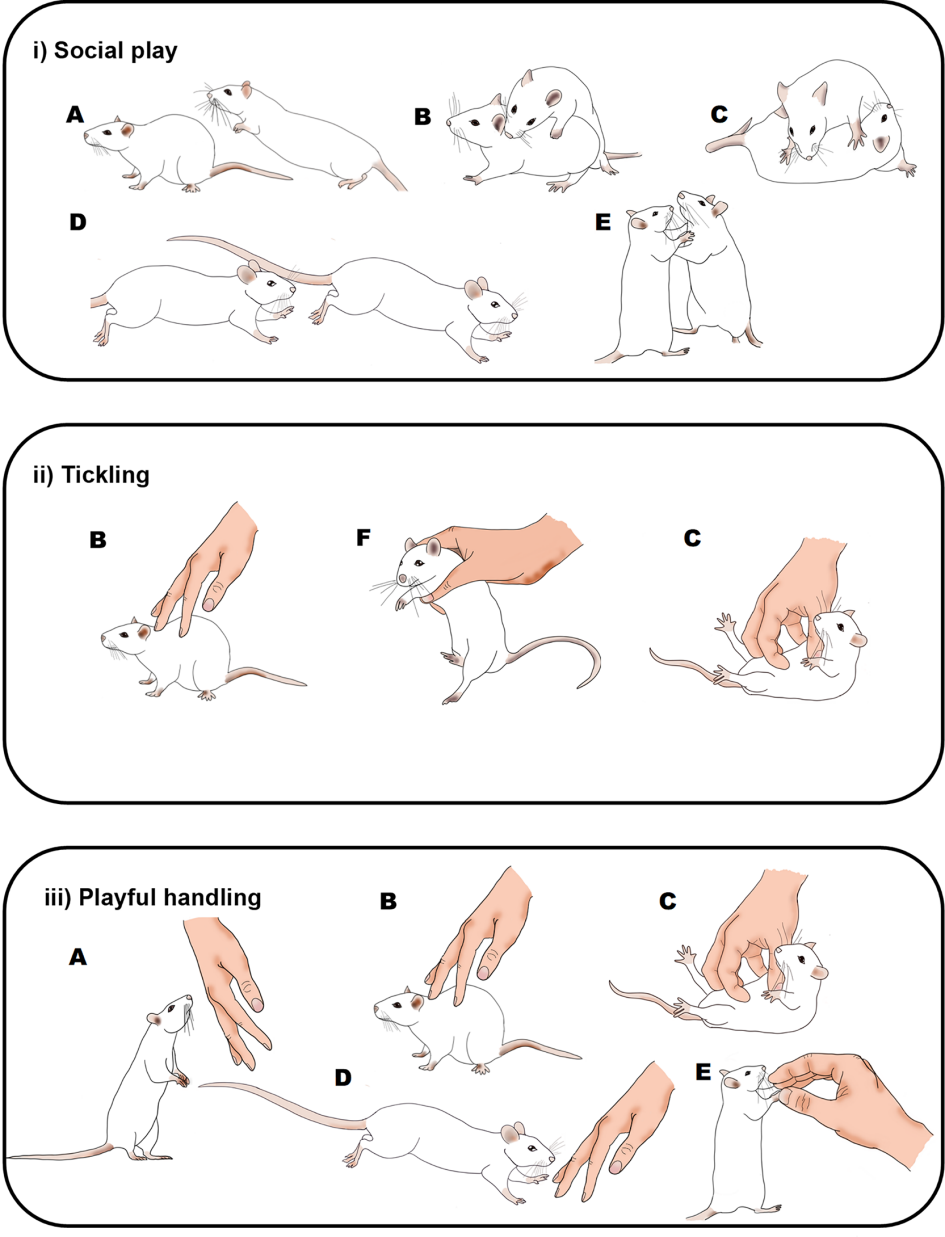
Graphical comparison of playful rat interactions.

During 2020 in the UK, 201,600 experimental procedures were carried out on rats, the second most investigated laboratory species ( UK Home Office, 2021). Worldwide there has recently been an increasing emphasis on positive welfare, and the introduction of positive experiences to improve animals’ quality of life ( Lawrence et al., 2019; Makowska & Weary, 2021). The use of manipulations to enhance positive welfare in laboratory animals has been shown not to be a source of variability ( Kentner et al., 2021), and a possible contribution towards improved model validity and data reproducibility ( Loss et al., 2021; Van De Weerd et al., 2004; Würbel & Garner, 2007). It is therefore likely that ‘happy animals make good science’ ( Grimm, 2018; Poole, 1997). For instance, positive welfare might reduce stress caused by aversive procedures ( Hinchcliffe et al., 2022). For laboratory rats, a positive handling approach referred to as rat tickling is proposed as an effective and practical approach to improve their welfare ( https://www.nc3rs.org.uk/rat-tickling ; https://na3rsc.org/rodent-handling/). Rat tickling with the human hand was developed to study positive emotions and ultrasonic vocalisations (USVs) in rats by mimicking playful interactions between rats ( Panksepp, 1998, Figure 1i). The standard tickling protocol involves initial finger contact with the nape of the neck before flipping the rat and gently pinning it in a supine position whilst making rapid finger movements focusing on the ventral surface as used in human tickling ( Cloutier et al., 2018; Figure 1ii).

Illustration of the behaviours seen in rats during: i) social play ( Pellis & Pellis, 2013); ii) the Panksepp variation of tickling ( Cloutier et al., 2018); and iii) playful handling ( Bombail et al., 2019, 2021). Letters indicate rat behaviours that share similar or the same physical characteristics: A) pouncing, B) nape contact, C) pinning, D) chasing, E) boxing and F) flipping. During social play (i) or playful handling (iii), behaviours do not always occur in each play bout; pinning (C), chasing (D) and upright (E) are almost always preceded by a nape attack or an attempted nape attack. During tickling, the sequence of behaviour is always B), F) and then C). Drawings by Tayla Hammond.

Research (see Bombail et al., 2021; LaFollette et al., 2017 for reviews) has shown that when rats are tickled by the human hand, they often produce 50 kHz USVs, which are understood to indicate positive affect in rats ( *e.g.*, Barker, 2018, Figure 2i). Other evidence suggests that rats find tickling rewarding as in addition to production of USVs, tickled rats show a reduced latency for approaching the human hand and can be trained to perform operant responses for tickling ( Burgdorf & Panksepp, 2001; LaFollette et al., 2017).

(i) Illustration of: (1) frequency modulated 50 kHz USVs, (2) flat 50 kHz USV and (3) 22 kHz USV; the spectrograms are taken from our own recordings and these vocalisations are typically expressed in all rats. Vertical axis represents USV frequency (in kHz) and horizontal scale bar represents 50 ms for USV 1 and 2, and 200 ms for USV 3.
(ii) Relationship between 50 kHz USV production and an affective bias test (redrawn with data available online, and reproduced with permission from Hinchcliffe et al., (2020)). Each data point represents, for each of the 16 rats, the 50 kHz USV response to tickling and the substrate preference in the Affective Bias Test, indicative of the degree of emotional valence.
(iii) USVs in playfully handled (PH) rats compared to a control group on the 1 ^st^ and 5 ^th^ session of treatment (redrawn with data available online and reproduced with permission from Bombail et al., (2019)). By the 5 ^th^ session, all PH rats showed an increase in USV production in contrast to panel (ii), which used the standard protocol where some rats show little or no increase in USVs.

**Figure 2.**
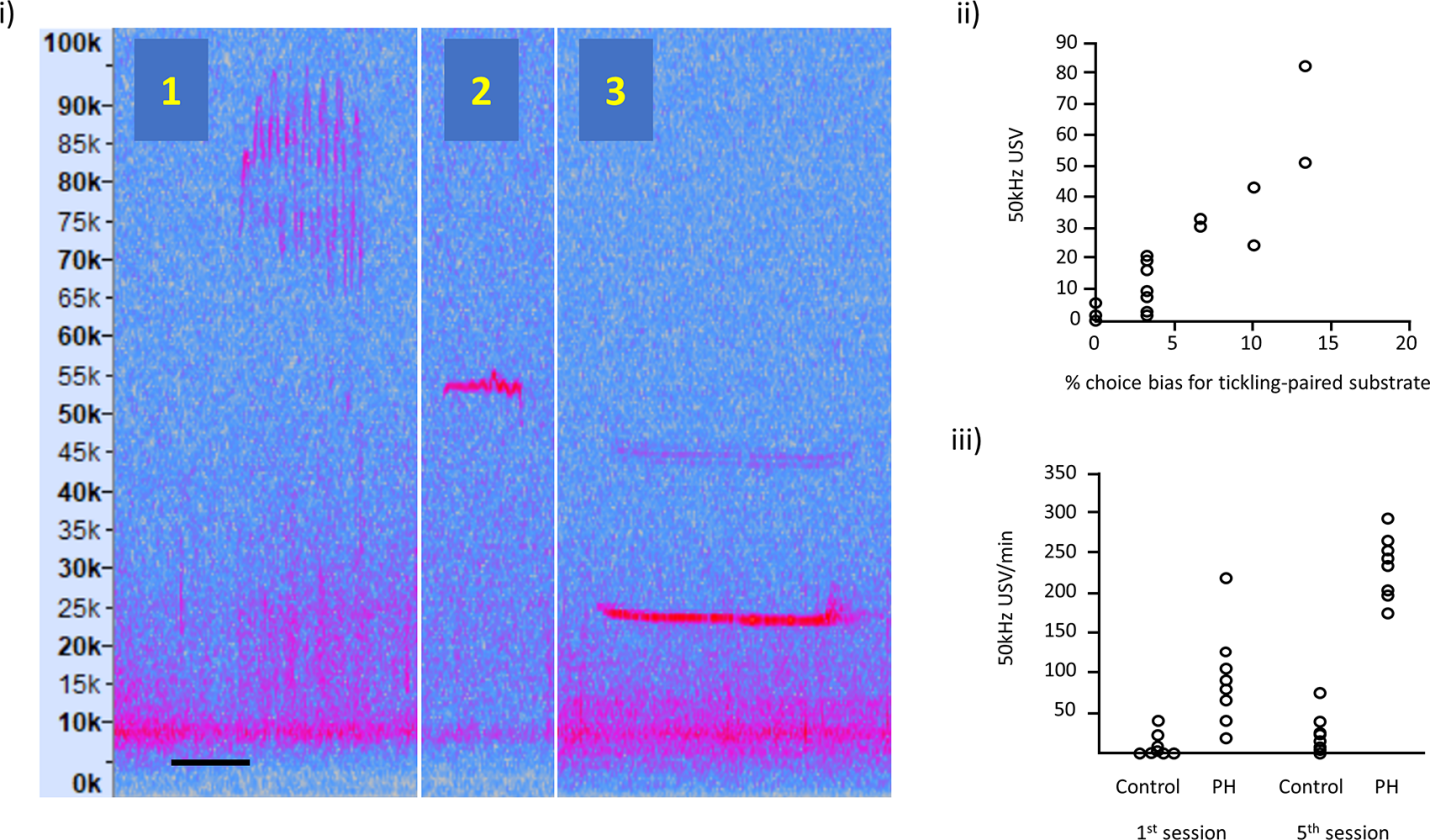
Examples of rat ultrasonic vocalisations (USVs).

However, individual responses to the standard tickling protocol indicate that there is a risk that some rats may not perceive tickling to be a positive experience. We summarise that evidence below (see also Bombail et al., 2021).

It is well accepted that rats vary in their response to tickling: The NC3Rs website on tickling is clear on this point: “But I hate being tickled; shouldn’t rats hate it too? This is a common concern, and it is true that some rats enjoy it more than others” ( https://www.nc3rs.org.uk/tickling-rats-improved-welfare ). We initially developed doubts over aspects of the standard tickling protocol ( Bombail et al., 2019) with its regular use of pinning due to the individual variation in USVs and behaviour we observed in response to this protocol ( *e.g.*, Hinchcliffe et al., 2020); see Figure 2(ii). Other work has reported significant levels of individual variation in response to tickling ( LaFollette et al., 2017).

Individual responses to tickling have welfare implications: Rat tickling response is associated with responses indicating affective states ranging from neutral to positive ( *e.g.*, Hinchcliffe et al., 2020), and hence tickling may not always be welfare enhancing. We have shown that USVs in response to tickling are correlated with an independent test of affective state (an affective bias test) indicating that rats vary significantly in their affective response to tickling ( Hinchcliffe et al., 2020). Figure 2(ii) taken from Hinchcliffe et al. (2020) shows variation in USVs in response to tickling correlated with the % choice bias for a tickling-associated digging substrate as the validated measure of affective state. Other evidence supporting a relationship between this naturally occurring variation in response to tickling measured as USV production and welfare outcomes, such as affective states, can be found in work from Rygula et al. (2012) and Mällo et al., (2007, 2009).

We therefore propose that individual variation in response to the standard tickling protocol is a significant issue when promoting the widespread use of tickling as an approach to improving rat welfare. Due to our experiences using the standard tickling protocol we have developed a refined tickling protocol we refer to as playful handling (PH), intended to better mimic the dynamic nature of play, giving the rat more choice over how it is tickled ( Bombail et al., 2019), Figure 1(iii). The PH protocol is described by Bombail et al., (2019) as the handler using one hand to touch, tickle, and play with the rat for 20 second periods interspersed with 20 second pauses. During the active periods, the handler mimicked the rough-and-tumble play seen in adolescent rats, with the hand tickling, chasing and pinning the rat, depending on their response. Our data suggest that the PH protocol resulted in a more uniform increase in USVs across individuals relative to the standard protocol. Figure 2(iii) adapted from Bombail and colleagues ( Bombail et al., 2019) shows USVs in playfully handled rats *vs.* a control group (who were not playfully handled) on the 1st and 5th session of treatment. By the 5th session, all PH rats showed an increase in USV production in contrast to Figure 2(ii), which used the standard protocol and where some rats show little or no increase in USVs.

(Panel i) Graphical representation of the experimental protocol. Upon reception, each cohort (n = 4 cages, with two males per cage and n = 4 cages, with two females per cage) was assigned to cage and treatment, then left to acclimatise for a week. Phase 1 consisted of exposure to six sessions (30 seconds daily) of either Control, P0, P1 or P4 treatment (the latter three varying in the number of pins administered relative to background playful handling; see Objective 1 above). Finally, the rats were behaviourally tested in Phase 2, using Elevated Plus Maze (EPM) then Open Field (OF), and killed for tissue collection. A step-by-step protocol is outlined in Box 1. (Panel ii) Prediction against which our confirmatory hypothesis will be tested: we predicted P0 and P1 treatments would lead to higher and less variable induction of ultrasonic vocalisation (USV) production, compared to P4.

Box 1. **Step-by-step protocol.**

Prior to the experiment and from Day 1 to Day 6 of the first cohort, various optimisation and preparation test recordings were carried out, this resulted in a similar level of background noise throughout the experiment (when several cohorts are housed and tested simultaneously)

Day 1. Reception of rats and group formation (morning).

Temporary mark on tail

Weighed each rat,

Formed eight cage pairs based on: (1) sex, (2) provenance (always housed with an individual from a different litter), (3) similar cage weight (monitor total treatment group average as the values must balance by the 8th cohort).

Day 1 to Day 6. Rats habituated to facility environment.

Day 7. Habituation to PH testing arena.

Collected faecal pellets for faecal corticosterone metabolite (FCM) analysis. Weighed rats. Habituation 1: in the morning (start of dark phase), for each of the eight cages, transferred pairs into the play arena and left together for five minutes. Removed any faeces from arena if necessary.

Habituation 2: in the afternoon, for each of the eight cages, transferred pairs into the play arena and left together for five minutes. Removed any faeces from arena if necessary.

Day 8. Habituation to the PH testing arena.

Preparation of the next cohort (Day 1)

Habituation 3: in the morning, for each of the 16 individuals, placed into the play arena and left for five minutes. Removed any faeces from arena if necessary. Habituation 4: in the afternoon, for each of the 16 individuals, placed into the play arena and left for five minutes. Removed any faeces from arena if necessary. Cage bedding is changed.

Day 9 to Day 11. Experimental treatment.

The cage moved close to the testing arena five minutes prior to the first of the pair of rats being introduced in the test arena. Both rats were filmed during this anticipatory phase, to investigate their behaviour.

From each cage, a rat was taken individually into the arena (order of passage pseudo-randomised), filmed for both a one min anticipation phase and treatment phase according to the schedule (control, PO, P1 or P4) for 30 seconds. Ultrasonic vocalisation (USVS) also recorded. Arena wood shavings were stirred between each animal (for odour homogenisation).

Following this treatment, rats were gently picked up and returned to the home cage. Their cage mate then immediately placed in the arena for testing and the same protocol as above repeated.

The home cage was covered with a cardboard screen, behind the plastic

curtains protecting other cages from emotional contagion via USV.

Two hours later, each cage was placed back on the housing rack.

Days 12 and 13 fell over the weekend, animals were undisturbed except for welfare checks.

Day 14 to Day 16. Experimental treatment.

Protocol as per Day 9 - Day 11

In addition, morning of Day 14 only: Weighed rats

In addition, Day 15 only: preparation of the next cohort (Day 1)

Day 17. Collect faecal pellets for faecal corticosteroid metabolite (FCM) analysis. Elevated Plus Maze (EPM) testing late morning (middle of dark phase). Standard EPM protocol, EPM will be cleaned between each animal.

Day 18. Open Field (OF) testing.

Rats were tested in the open field (OF) in the morning. Standard OF protocol, OF will be cleaned between each animal.

Rat weighed

Rats injected with an overdose of pentobarbital. Cage order was randomised and rats within a cage were killed sequentially. Final faecal pellets were collected for FCM analysis by taking the pellet closest to the colonic flexure. Exsanguinated blood and tissues (heart, liver, caecum, brain, kidney) samples were collected for current and future molecular studies to investigate physiological impact of experimental treatments. Further description of these can be found in section 2.7.

We conclude that this evidence calls for a scientifically based refinement of tickling to ensure that the positive effects of tickling are maximised and that individual differences in response to tickling are minimised with benefits to rat welfare and the reproducibility of research where tickling is applied as a treatment or more generally as an enrichment. In this report, we define tickling as the standard protocol ( *e.g.*, Cloutier et al., 2018) and, although it was initially coined as an equivalent term to tickling ( Cloutier et al., 2018), PH as the approach outlined in Bombail et al. (2019). ^1^

### 1.2 Hypotheses

PH consists in mirroring playful behaviour by allowing for more flexibility between the human handler and the rat and consequently we hypothesised that PH would induce a more homogeneous positive affective response. We also hypothesised that PH would lead to reduced variability of biological outcomes.

*Confirmatory Hypotheses:* ( *Confirmatory Hypothesis* 1) We hypothesised that rats exposed to handling protocols that maximise playful interactions ( *i.e.*, where pinning number per session decreases) would show an overall increase in total 50 kHz USVs as an indicator of positive affect. ( *Confirmatory Hypothesis* 2) We also predicted that 50 kHz USV measures would be less variable among individuals in treatments with less pinning.

*Exploratory Hypothesis 1:* We hypothesised that the behavioural and physiological changes associated with alterations in PH experience would be less variable among individuals when rats are exposed to handling protocols that maximise playful interactions (where pinning number per session decreases). There are no previous data on this question, so we cannot generate power calculations and pre-register this hypothesis, this is therefore exploratory work.

### 1.3 Study timeline

**Figure 3.**
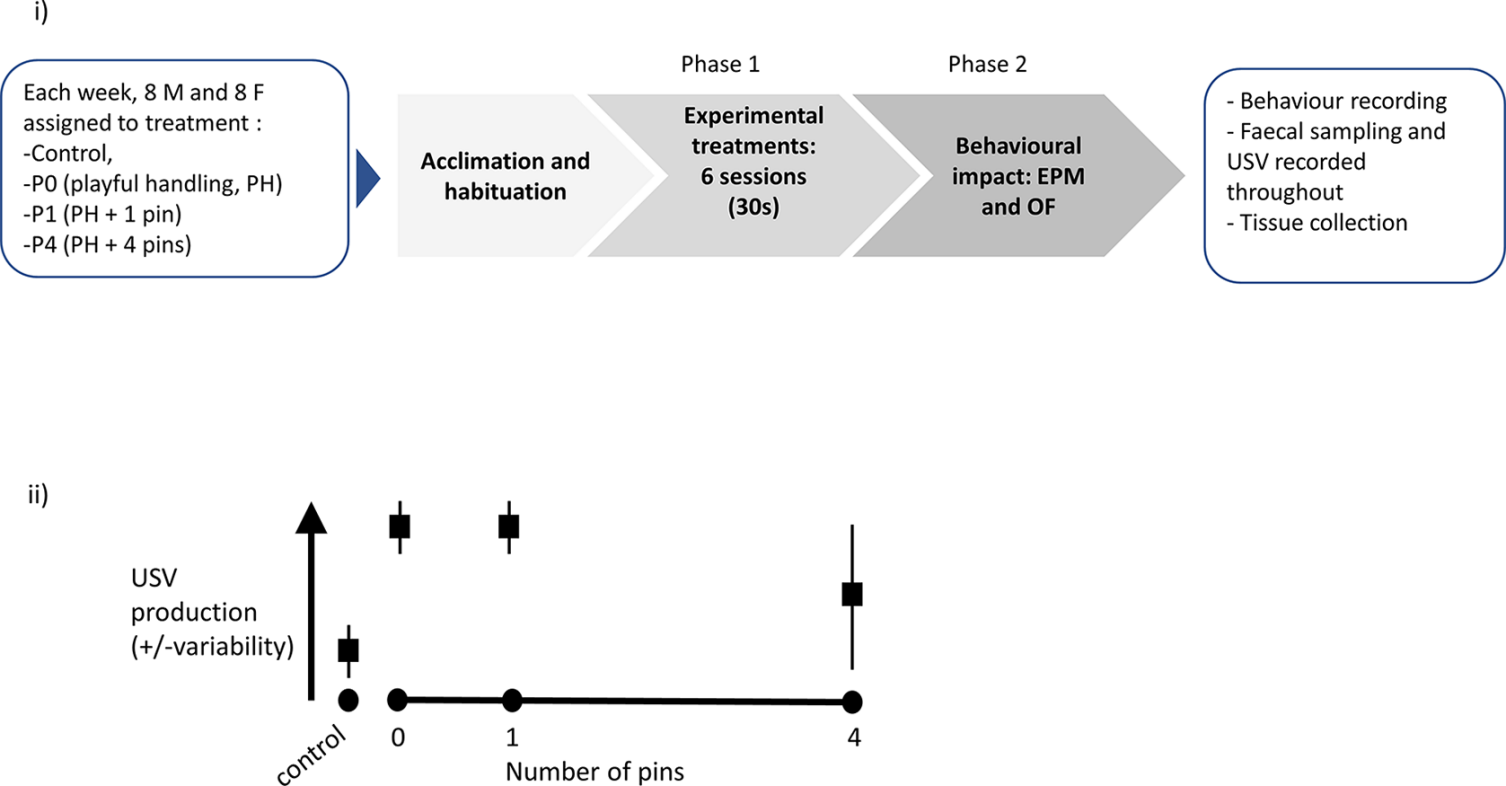
Experimental plan and graphical illustration of hypothesis.

The diagram presented in Figure 3 synthesises the timeline for each three-week long cohort studied. The experimental part of this study was conducted using eight such cohorts, each made up of 16 animals. The timing of these cohorts was staggered, so experimental cycles overlapped, with 16 animals brought into the facility every week. A step-by-step protocol is available in the Stage 1 registered report and is also presented below ( Box 1).

## 2. Methods ^2^

### 2.1 Basic methodological framework/identification strategy

Our experimental design was a pseudo randomised control trial where every week, each of the eight cages of two male or two female rats would be assigned to one of four treatments (control, P0, P1, P4). Within a cage, individuals cannot be considered independent, as they might influence each other in response to the treatment through emotional contagion. Therefore we fitted the cage as a random effect for all treatments and both sexes.

### 2.2 Ethical approval

All animal work was carried out at the Roslin Institute, Edinburgh, U.K., in accordance with the U.K. Animals (Scientific Procedures) Act 1986 and reported in compliance with the ARRIVE guidelines ( Bombail, 2022). This work did not involve aversive procedures (and is deemed less harmful and stressful than skilled insertion of a hypodermic needle according to good veterinary practice) and therefore not legally required to have UK Home Office approval. As recommended ( Olsson et al., 2022), we collectively discussed welfare and ethical considerations with the Named Animal Care Welfare Officers, veterinarians and scientists involved in the study. Following consideration by the University of Edinburgh’s Animal Welfare and Ethical Review Body (reference NC/W001209/1, 26 July 2022, Establishment number X212DDDBD), it was confirmed the work was subthreshold to regulation under the U.K. Animals (Scientific Procedures) Act 1986, and the committee approved it as detailed in this paper. This study was also approved by the Veterinary Ethical Review Committee of the Royal (Dick) School of Veterinary Studies, University of Edinburgh (reference 92.22, 2 September 2022).

### 2.3 Experimental animals

Practical and financial constraints required that we limit this study to a single strain. We used Wistar rats (Research Resource Identification, RRID:RGD_2308816) purchased from Charles River Laboratories (CRL, Margate, UK). This outbred albino strain has been shown to be reliably responsive to tickling and PH ( Bombail et al., 2019; Hammond et al., 2019) and is one of the commonly used strains for such experiments (23% of rat tickling studies used Wistar rats; LaFollette et al., 2017). We purchased the rats from CRL (16 at a time), aged 3-4 weeks and completed the research prior to their puberty around 7 weeks of age as over 60% of papers on tickling used rats within this time window ( LaFollette et al., 2017). We have also recently found evidence of sex differences with female rats producing more USVs than males in response to tickling ( Tivey et al., 2022).

We carried out the experiment in eight cohorts of n = 8 cages (with two rats per cage) with equal numbers of males and females in each cohort. A total of 128 rats (64 males and 64 females) were used in this study, in accordance with our power analysis detailed below.

To the greatest extent possible we aimed for a matching number of males and females coming from the same litter. The number of litters sampled were two per cohort (four males and four females from the same litter) with no repeat matings across cohorts. Rat treatment allocation was spread evenly across all litters to prevent bias due to genetic background and early life experience ( *i.e.*, each of the four individuals from each sex was assigned to each of the four treatments). Only animals from experienced parents (sire/dam will have been sired/nursed at least one previous litter) were used. This reduced variability in the early life environment for the pups in case of effects of differences in parental experience.

### 2.4 Acclimation, treatment allocation, housing and husbandry

On arrival at the Roslin Institute facility, the rats were housed in a bespoke behaviour research room with an average room temperature of 22°C and relative humidity of 43 %. The rats were on a 12 h: 12 h reverse light cycle (lights off at 06:00), to allow testing of the rats during their dark photoperiod when they are most active and to accommodate the preference of albino rats for lower light intensities (see Hammond et al., 2019). All treatments and behavioural tests were carried out between 08:00 and 16:00 (2 hours from lights on or off ). The allocation of rats to cages was carried out in the following order: sex, litter of origin and body weight. Rats were weighed and allocated to their home cages as pairs of the same sex (but never from the same litter). Upon assignment to cages, the heaviest sex-matched rats from each litter were housed together, and subsequently the second, third and fourth heaviest together. As much as feasibly possible, we aimed to house together rats of similar weight. Pairs were assigned to a treatment using a random list generator ( https://www.random.org/lists/). We aimed to homogenise weights between groups. For each sex, rat weight was averaged for each treatment, and to avoid potential bias caused by uneven distribution of body weight between treatment groups, adjustments in treatment distribution were made to balance evenly weights across treatments ( *e.g.*, we assigned heavier pairs of animals to treatments that previously contained lighter animals). Rats were then left to acclimatise for six days prior to handling.

The home cages are made of clear plastic with a metal mesh open-top lid (55 cm x 38 cm x 21 cm: L × W × H), filled with 2 cm deep wood chip bedding, shredded paper, a 20 cm long red plastic tunnel, a 10 cm wooden chewstick and a 2 cm diameter wooden ball as enrichment (all as per normal practice in our animal facility). This size cage allowed animals to rear and stand on their hind limbs. Rats had *ad libitum* access to standard rat chow (current supplier: Teklad Global Rodent Maintenance Diet (14 % protein); Envigo, UK) and tap water. The cages were distributed across four tiers of a standard cage rack. Due to the varying lux levels and other potentially confounding variables, position of the home cages in the rack was randomised across treatments (see Hammond et al., 2019). The rats were checked daily, and nitrile gloves worn when handling the animals. To minimise handling stress, rats were picked up gently by holding them behind their forelegs and then cupping them with both hands. As cage size was larger than we initially planned, and larger than recommended laboratory animal guidelines, in agreement with the facility staff we decided to remove the potentially stressful cage change events and let the animals live in the same litter for the duration of the experiment (we visually confirmed it was not overly soiled at the end of the experiment). Body weight was measured routinely across the study (on days 1, 7, 14 and 18).

### 2.5 Experimental treatments

A unique male experimenter (with prior experience in all rat playful interactions tested) performed all treatments and behavioural tests. All experiments were performed in the same room in which the animals are housed, as in previous studies ( *e.g.*, Hammond et al., 2019). Using curtains and physical distance, we prevented emotional contagion phenomena involving USV ( *i.e.*, we have confirmed high frequency 50 kHz USV cannot be detected by other animals, using our recording equipment). Rats were used to a degree of audible activity in the room throughout the experiment. The play arena, placed behind fabric curtains 6 m away from the home cages, was a Plexiglas square (60 cm x 60 cm) of depth 25 cm, filled with 3 cm-deep woodchip litter in which Wistar rats had played previously (over 200 sessions by 16 males rats took place prior to the start of the experiment). We kept the same litter for all experiments and removed any faeces. In our experience, faecal pellets are exclusively produced during the habituation sessions. Between tests, the bedding litter was regularly stirred. We used this method to guarantee the litter contained odorants from several rats, thereby standardising, diluting and minimising the impact of individual olfactory cues. This could be replicated in other laboratories by using a mixture of soiled litter and clean litter. Following experimental treatment (described below), cages were left, loosely covered with cardboard screens, for approximately 2 h in an area behind plastic curtains (a switched-off Lateral Air Flow cabinet), before being returned to their usual home cage rack position. In the absence of this ‘cool down’ period, in our experience a marked rise in USV production over time can otherwise be measured in control rats ( Lam, 2017).

The rats were habituated to handling and the experimental recording arena, to lessen any anxiety or fear due to novelty, as negative affective states inhibit play behaviour (reviewed in Ahloy-Dallaire et al., 2018). For habituation to the treatment arena, rats were moved in their cage to be beside the treatment arena before being lifted gently into the arena, and left there to explore, first as a pair of cage mates (2 × 5 minutes), on the first day, and then alone (2 × 5 minutes) on the second day.

The aim of PH is to give all rats a similar amount of hand contact (we approximate 70 – 80 % of human-animal interaction time, *e.g.*, about 24 out of 30 seconds), while being responsive to differences between individuals. The similar amount of physical contact for all individuals is important, since activation of the cortical area in response to touch has been shown to influence USV production ( Ishiyama & Brecht, 2016). When not touching the rat, the hand can also approach the animal while the hand is in contact with the wood shavings, to provide sensory cues consistent with a play partner approaching ( *e.g.*, about 6 out of 30 seconds).

Another key component of PH is to be responsive to individual differences, which can range from more playful individuals who will quickly move (accelerate or turn) to face the hand, and display playful behaviour ( *e.g.*, joy jumps Reinhold et al., 2019) to less playful individuals who move less and more slowly and show little or no play behaviour. An example of PH with a responsive individual is available to watch online here ( https://www.youtube.com/watch?v=awwcKVe14ZI). PH starts with a presentation of the back of the experimenter’s hand for a brief sniff and exploration by the rat. The experimenter then starts playing with the rat using a rapid tickling finger motion on the sides, back and front of the rat (alternating region). The experimenter should focus on being playful (unexpected moves, fast change of hand movements, change of pace) rather than simply the physical act of tickling ( *e.g.*, Figure 1iii).

Pinning consists in approaching the rat during PH as described above. Thumb and middle fingers are then placed behind the front legs, and the animal is gently but swiftly moved onto their back, and upon landing, while the rat is pinned down, s/he is tickled on the front of their thorax and abdomen involving rapid tickling movements of fingers ( *e.g.*, see Cloutier et al., 2018).

In both PH and standard tickling, movements should reflect that rat play is boisterous ( Cloutier et al., 2018) with finger pressure being firm and movements fast.

### 2.6 Objectives

**2.6.1 Objective 1: Testing protocols varying in PH and pinning events against USV as a measure of affective state**

*Confirmatory Hypotheses:* (Confirmation Hypothesis 1) We hypothesised that rats exposed to handling protocols that maximise playful interactions (i.e., where pinning number per session decreases) would show an overall increase in total 50 kHz USVs as an indicator of positive affect. (Confirmation Hypothesis 2) We also predicted that 50 kHz USV measures would be less variable among individuals in treatments with less pinning.

Objective 1 was addressed in Phase 1 of the experiment (see Figure 3).

*Experimental design:* The aim of this objective was to refine the standard tickling protocol based on the rats’ affective response to treatments that vary in PH and number of pins. All rats were exposed to six sessions of experimental human-animal interaction treatment, over eight days, interrupted by a two-day weekend break. We have chosen a standard session length of 30 seconds that reflects our previous work ( Hinchcliffe et al., 2020) and also the practical reality that if tickling is to be used widely in practice it needs to be applied in a time efficient manner ( LaFollette et al., 2019). The order of testing of cages, and rats within cage were pseudo-randomised (a sequence for cage and individuals generated using a random list generator). The cage was moved close to the testing arena five minutes prior to the first of the pair of rats being introduced in the test arena. Both rats were filmed during this anticipatory phase, to investigate their behaviour.

Rats were removed individually from the home cage, placed into the handling arena and, following an additional one-minute recording of anticipatory play behaviour, the experimental treatments applied:

P0: ‘PH’, where zero pins were applied; the experimenter used one hand to touch, tickle and chase the rat in a manner that mimics rough and tumble play ( Bombail et al., 2019); Figure 1(iii).

P1: One pin was applied at approximately 15 seconds from the start of the tickling session. The rat was held and flipped into a supine position and the experimenter gently pinned the rat in this position whilst moving their fingers quickly and vigorously but gently on the belly, as is commonly used in human tickling. The pin lasted for between 2 – 4 seconds and the rat was allowed to right themselves at the end of pinning ( Cloutier et al., 2018); Figure 1(ii). On either side of the pin the experimenter engaged in PH as in P0.

P4: Four pins were applied from five seconds into the tickling session (four pins being the number delivered during a 15 second pinning session in the standard protocol ( Cloutier et al., 2018)); pins were applied as in P1 and the rat was allowed to right themselves after each pin with a short gap before the next pin. On either side of the pinnings the experimenter engaged in PH as in P0.

Control: We applied the same control treatment as in our previous work ( Bombail et al., 2019; Hammond et al., 2019) where the experimenter placed their hand against the inside of the arena for the entire session.

Following this treatment, rats were gently picked up and returned to the home cage. Their cage mate was then immediately placed in the arena for testing and the same tickling treatment repeated.

**2.6.1.1 Data collection**

Behaviour during the tickling sessions were recorded using a digital HD camcorder. USVs were recorded using a high-quality microphone designed for recording USVs produced by bats (Pettersson M500-384 USB Ultrasound microphone) and Audacity recording software ( http://www.audacityteam.org/). The microphone was placed at a 40 cm distance towards the centre of the arena, pointing downwards at the arena floor. Total USV per unit of time (minute) was quantified by spectrogram analysis. ^3^

Measures: (i) Vocalisations were recorded during the handling sessions as in our previous work ( Bombail et al., 2019; Hammond et al., 2019) and categorised into 50 kHz USVs and 22 kHz USVs; (ii) We recorded other quantitative behavioural responses to tickling as per our previous work ( Hammond et al., 2019).

Our primary response variables for Confirmatory Hypotheses 1 and 2 were:

(a) Total 50 kHz USV: We analysed 50 kHz USV as indicators of positive affective state ( *e.g.*, Barker, 2018; Brudzynski, 2013). We defined 50 kHz USV as calls with a peak frequency between 30 and 80 kHz and a short duration between 10 – 400 ms (based on ( Brudzynski, 2013) and our own empirical observations). We predicted 50 kHz USV would be significantly increased in P0 and P1 treatments, where pinning is minimised, relative to P4, where play is restricted relative to pinning, and the Control treatment, where there is no play.
(b) Variability of 50 kHz USV production: We compared individual variability across the different treatments. We predicted reduced among-individual variability in USV production in treatments that maximised playful handling.

Our secondary response variables were:

(a) Categorisation of 50kHz calls: We will analyse sub-types of USV following segregation based on their acoustic properties, according to published criteria ( Wright *et al.*, 2010). We will investigate whether certain USV types might be preferably associated with certain treatments.
(b) Anticipatory solitary play behaviours ^4^: We measured anticipatory play behaviour, as this has been shown to be an indicator of positive affect. We will use the ethogram in Table 1, based on observations from our earlier work ( Bombail et al., 2019; Champeil-Potokar et al., 2021; Hammond et al., 2019).
(c) Behavioural transitions: We analysed the number of behavioural transitions (including play) that occurred prior to treatment, as indicators of reward anticipation ( Anderson et al., 2015).
(d) 22 kHz USV: We also pre-registered analysing 22 kHz USVs as indicators of negative affective state ( Brudzynski, 2013). We defined 22 kHz USV as calls with a frequency between 20 – 30 kHz, and a long duration between 500 – 3000 ms ( Brudzynski, 2013). We predicted very low numbers of events overall, and expected that they clustered in treatments that maximise pinning. ^5^

**Table 1.** Ethogram for study of anticipatory behaviour. Based on our previous studies and adapted from ( Bombail et al., 2019; Champeil-Potokar et al., 2021; Hammond et al., 2019). ^11^

Behaviour	Description
Move	Locomotor behaviour, e.g., mid rearing or going down, or travelling horizontally
Up explore	Sniffing and exploring, while rearing
Down explore	Sniffing and exploring, interaction with litter, eating (litter or coprophagia)
Dig litter	Dig litter
Immobile	Lack of perceivable movement
Grooming	Includes partial grooming sequences
Solitary play	Seemingly spontaneous movement involving at least two hops, where a hop involves all four paws leaving the ground at the same time; can occur from stationary or during locomotor movement; behaviour performed not in the direction of the cage mate (play partner) during a play bout or as an evasion response to being chased by a play Described in Figure 1c. One rat pounces or rubs on the partner to solicit play, resulting in the partner either chasing the soliciting rat, rearing (the pair makes rapid pawing movements at each other) or rotating to where one rat is pinned onto its back with the other standing over it.
Social play	Described in Figure 1c. One rat pounces or rubs on the partner to solicit play, resulting in the partner either chasing the soliciting rat, rearing (the pair makes rapid pawing movements at each other) or rotating to where one rat is pinned onto its back with the other standing over it.

**2.6.2 Objective 2: Testing the behavioural and physiological effects of protocols varying in PH and pinning events**

*Exploratory Hypothesis:* We hypothesised that the behavioural and physiological consequences of tickling would be less variable among individuals, and more repeatable when rats were exposed to handling protocols that maximise playful interactions and where pinning episode number per session decreases. Due to the paucity of data on the impact PH might have on the variables we will measure, we could not generate power calculations and pre-register this hypothesis, this is therefore exploratory work.

Objective 2 was addressed in Phase 2 of the experiment (see Figure 3).

*Experimental design* ^6^ *:* In Phase 2, the next day after the final playful or control interaction, rats underwent behaviour testing with Elevated Plus Maze (EPM) and Open Field (OF), their order of passage was pseudo-randomised. The EPM and OF test consist in quantifying exploration behaviour in 2 different structures, that both consist in an area with open spaces (the centre of the OF and the open arms of the EPM) and an area with more enclosed spaces (the periphery of the OF and the closed arms of the EPM). For rats, this ambiguity creates a conflict between the thigmotaxis (preference for proximity to walls) and the desire to explore open spaces, which was used to assess the emotional response to those novel experiences, and to test for anxiety levels. Following these tests, the rats were humanely killed by anaesthetic overdose and tissues collected for analysis. Post mortem tissue measures were critical to assess the impact of our treatments on variability and repeatability of commonly used measures of physiological function. Faecal pellets were collected on days 17 (morning) and 18 (post cull) for non-invasive corticosterone metabolite analysis, in comparison to pellets collected before treatment starts on day seven.

**2.6.2.1 Data Collection**

*Measures* ^7^ *:* (i) Behavioural measures of response to OF and EPM ( Ethovision) were collected to quantify anxiety behaviour prior to culling; (ii) Physiological measures of stress and inflammation: Faecal samples were collected on day 18, at the end of Phase 2 (and compared to levels at the start and end of Phase 1) and assayed for corticosterone ( Lepschy et al., 2007) using a commercially available corticosterone ELISA (Enzo LifeSciences). Point of cull plasma was assayed for a set of inflammatory markers including the pro-inflammatory cytokines IL1a, IL1b, IL2, IL6, IFNγ and TNFα and the anti-inflammatory cytokines IL4, IL10, IL13, using a magnetic bead based multiplex ELISA (FIRELEX); our selection of inflammatory markers included cytokines known to be sensitive in humans to negative and positive affect ( Anisman & Merali, 2003; Stellar et al., 2015); (iii) Other tissues such as the brain, pituitary gland, heart, liver, spleen and gut were collected at culling and flash frozen on dry ice and stored at -80°C.

Our primary response variables were:

a) EPM: Increased markers of open arm exploration (% time and distance exploring the open arm) reflect reduced anxiety, based on rats’ aversion of open spaces ( Kraeuter et al., 2019).
b) OF: Increased markers of thigmotaxis (% time spent and distance covered close to the peripheral walled area) reflect increased anxiety, based on rat aversion of open spaces. Markers of activity (rearings, total distance covered, bouts of locomotion) were also recorded since, although interpretation can be challenging ( Denenberg, 1969), they do reflect arousal upon OF arena exploration.

Our secondary response variables were:

a) Physiological and inflammation related variables: We analysed markers regulated in affective states, and their variability around the treatment mean/median. We expected P0 and P1 to be more effective at inducing anti-inflammatory cytokines and reducing pro-inflammatory cytokines, and FCM ( Anisman & Merali, 2003; Lepschy et al., 2007; Stellar et al., 2015), in comparison to P4 and control treatments.
b) Body weight: We kept a record of weight as an indicator of general body condition, we did not expect any treatment-related changes, as per our previous studies ( Bombail et al., 2019; Hammond et al., 2019).

### 2.7 Statistical analyses ^8^

Using the same approach as described for Objective 1, data will be analysed in R using Generalised Linear Mixed Models to ascertain the effects of tickling treatments and sex on behavioural and physiological measures. To test our Exploratory Hypothesis that the biological consequences of tickling will be less variable among rats and more repeatable within rats when rats are tickled using more playful protocols (P1, P0), we will analyse the residual variance from the undertaken models and explore intraclass correlation coefficients of combined random and fixed effects

### 2.8 Diagram of the experimental plan

A diagram of the experimental procedure is presented in Figure 3i. We also present a graphical representation of our predicted results, which were tested in our experiments ( Figure 3ii).

### 2.9 Further exploratory analyses

Brain tissue was collected for future analysis of neurobiological consequences of the handling treatments. When funding and collaborations can be raised, it is our intention to further explore the biology of positive affective states through analysis of physiological functions that are associated with emotional experiences ( *e.g.*, immunology, gut microbiota, stress resilience markers such as HPA axis function or metabolism). We will also analyse USV production in relation to specific USV types ( Wright et al., 2010) using spectrograms from our recordings, with a view to correlating specific USV types to treatments and behaviours.

## 3. Results

### 3.1 Alteration in the experimental procedure

As required in Stage 2 reporting, we could not amend our protocol written at the time of Stage 1 publication. Here we describe changes to our original methods which we made either as improvements to our original plan, where some analyses could not be carried out as planned, or to rectify minor errors in our original submission.

**3.1.1 With reference to variability (from section 1.1)**

As an addition to the Stage 1 report, we wanted to be clearer on referring to individual variation in USVs as either occurring among individuals or within-individuals, in order to better understand the balance between stable individual differences and day-to-day fluctuations in vocalisations. These two components of variability offer complementary insights into how rats respond to treatments and how these responses may differ across individuals or change over time. For example, if some treatments fail to evoke a positive affective state in all rats, we expect that rats who prefer the treatment to produce significantly more USVs than those who do not. We would then expect an increase in among-individual variance, as the responses of different rats become more distinct. On the other hand, within-individual variance captures how consistent or variable a rat's USV production is over time. If a treatment increases within-individual variance, it may suggest that a rat’s preference for the treatment fluctuates across sessions, indicating a less stable or context-dependent response. The repeatability statistic measures how much of the total variation in USV production is due to consistent differences between individuals, as opposed to random or within-individual variation. This multi-factor perspective is important for interpreting the effects of treatments on behaviour as by identifying consistent individual differences, temporal fluctuations and overall repeatability, we can uncover the underlying factors driving these changes and design interventions that maximise positive welfare outcomes for all rats. In what follows, we have added text additional to the Stage 1 report to be more specific about the variability component we are referring to. These changes are therefore a clarification of our hypotheses on the effects of tickling treatment on individual variation.

**3.1.2 Protocol description in Box 1**

- On day 1: We changed the protocol from using a temporary mark on tail, to each individual retaining their experimental ID code throughout the experiment.
- Day 9 to Day 11, and Day 14 to Day 16. Experimental treatment: We now realise our wording (‘anticipatory’) was not adequate (in our protocol anticipation refers to the minute spent in the Perspex tickling arena prior to treatment administration). We should have referred to this as ‘cage anticipation’. However, in the event this is a redundant point as we did not analyse cage behaviour of the pair.
- Addendum to method for days 17 and 18:
  Day 17. Elevated Plus Maze (EPM) testing late morning (middle of dark phase) was carried out under red light. The EPM consisted of 2 open arms (L: 100 cm, W: 10 cm each; 337 lx measured on the runway) which intersected at 90° to form a plus shape with 2 closed arms with black walls of the same dimensions elevated 70 cm above the floor. The test was initiated when a rat was placed in the same open arm of the plus maze and allowed to explore freely. Tests lasted 5 minutes in duration and were digitally recorded from above using an infrared camera. Between testing each rat, the apparatus was cleaned with 70% ethanol and dried. Digital analysis of EPM was performed under Ethovision XT (Noldus).
  Day 18. In the Open Field (OF) testing, rats were tested in the open field (OF) in the morning under red light. The OF consisted of a plexiglass box measuring 100 cm × 100 cm × 25 cm (L × W × H) with clear sides and an opaque floor. The test was initiated when the rat was placed in the centre of the field and left to explore. Tests lasted 5 minutes in duration and were digitally recorded from above using an infrared camera. Between testing each rat, the apparatus was cleaned with 70% ethanol and dried. Digital analysis of the OF was performed under Ethovision XT (Noldus).

**3.1.3 Update of Section 2.6.1.1, to reflect changes in USV analysis**

- Total USV count per unit of time (minute) were quantified by spectrogram analysis, using DeepSqueak ( Coffey et al., 2019), instead of the manual analysis we initially intended (also see below).
- Description of secondary response variables: Categorisation of 50 kHz calls: we analysed sub-types of USV following classification based on their acoustic parameters from the DeepSqueak output. This is a change from our pre-registered report which proposed manual classification, according to published criteria ( Wright et al., 2010). Due to time constraints, we were not able to manually categorise USVs identified in this study. DeepSqueak allows extraction of USV acoustic properties such as Delta Frequency (indicative of Frequency Modulation) and Sinuosity (distinguishing flat vs non-flat calls), as described in Coffey *et al.* (2019).
- Home cage pair behaviour (incorrectly refered to as anticipation in the Stage One paper) was not analysed. Anticipatory behaviour in the tickling arena, in the minute preceding treatment administration, was analysed (solitary play behaviour occurred too rarely to analyse).
- 22 kHz analysis: In our dataset, these events were too rare to analyse.

**3.1.4 Amended ethogram for the behavioural analysis during the anticipation phase**

**3.1.5 Update on methodology for behaviour and tissue analyses**

**Ultrasonic vocalisations (USVs)**

USVs collected during the anticipation and treatment phases were analysed using the publicly available DeepSqueak program ( https://github.com/DrCoffey/DeepSqueak) running under Matlab (.mat) on licence to the University of Edinburgh. The DeepSqueak program provides outputs of total counts of vocalisations within these parameters, in addition to pre-selected spectral properties (sinuosity and delta frequency selected here). Data were exported into Excel and total counts transformed into rate per minute for analysis, to account for any minor differences in session length. To validate the accuracy of the DeepSqueak identification, Bland-Altman analysis ( Altman & Bland, 1983) was used to investigate the agreement between the DeepSqueak output and the accepted gold standard, manual identification by experienced personnel, on a proportion (64 out of 728 recordings) of the audio recordings. We found strong concordance between methods without evidence of systematic bias (average of USV count difference between methods was -3.8 ± 15.8 USV, data points were within 1.96 standard deviations and exhibited no bias).

**Analysis of Play behaviours and Behavioural transitions**

Video recordings from the anticipatory phase were played back at 0.5 speed and continuous behavioural recording performed. Behaviours were scored based on the ethogram ( Table 2). Behavioural transitions were reported as the total count of the number of times an individual changed their behaviour (transitioned from one behaviour to another, including pauses in repeated behaviours) within the observation period.

**Table 2.** Amended ethogram for the behavioural analysis during the 1 minute pre-treatment anticipation phase in the tickling arena.

Behaviour	Definition	State (with duration) or Event (instantaneous)
**Moving**		
Locomotion	Rat traveling in the cage horizontally.	State
**Exploring**		
Up-exploring	Sniffing and exploring, while rearing or head pointing upward.	State
Supported-rearing	Rat rearing up while their forepaws rest against the cage walls.	State
Down-exploring (Mobile)	Sniffing and exploring, with noise applied to any part of the cage or bedding material.	State
Fixed-sniffing	Rat is sniffing in a fixed position with no hind leg movement for at least 1 second.	State
**Fixed**		
Immobile	Lack of perceivable movement for at least 1 second.	State
Burrowing	Rat uses the muzzle or forepaws to dig or move the bedding materials. To be certain, the muzzle or forepaws must be invisible because of bedding material covering.	State
Self-Grooming	Rat performs various maintenance behaviours: face washing with forepaws, coat cleaning with muzzle, scratching with hindlegs.	State
**Vocalization**		
Audible vocalisation	Rat exhibits vocalization that is recognizable by recording device and human ears.	State
**Play**		
Solitary play	Rat performs seemingly spontaneous movement involving at least two hops, where a hop involves all four paws leaving the ground at the same time; can occur from stationary or during locomotor movement.	State
**Escape**		
Escape attempts	Rat jumps and put their forepaws on the wall edge, while hindlegs have no contact with the bedding ground.	Event
Wall-Climbing/Running	After escape attempt, rat puts their forepaws on the edge of the cage wall, while their hindlegs are contacting the wall or also on the wall edge.	State

**Analysis of faecal samples**

Faecal pellets were collected at three timepoints as indicated above and stored at -80 °C. Rat ID 13-1 (Female – Control) failed to provide a sample for timepoint 2. Frozen pellets were weighed and 100 ng transferred into a clean 1.5 ml Eppendorf in 1 ml absolute methanol. Samples were agitated and incubated overnight at 4 °C, followed by further agitation by vortex. Samples were then centrifuged at 2500 rpm for 10 minutes and the supernatant containing metabolites transferred to a clean 1.5 ml Eppendorf. Total protein concentration was determined using a DeNovix DS-11 spectrophotometer.

Sample dilution curves were used to determine the optimal loading concentration of sample ensuring that they stayed within the assay parameters. A sample concentration of < 0.1 mg / ml total protein was ascertained to be optimal, as such all samples were diluted to 0.1 mg/ml with assay buffer. Given the range of concentrations in the samples, this gave a dilution of between 1:40 and 1:100. Samples and standards were run in duplicate with one plate holding 32 samples. The faecal corticosteroid assay utilises an antibody for metabolite fractions containing 5a-pregnane-3b,11b,21-triol-20-one, first described by Touma et al. (2003). In brief, samples and standards were incubated overnight on anti-rabbit IgG coated plates with antibody and enzyme label solutions at 4 °C. The following day, plates cycle through multiple wash and incubation cycles, first incubating with streptavidin for amplification then a peroxidase solution for colouration. Oversaturation of sample wells was initially an issue so to prevent this the duration of the colouration step was reduced from 45 minutes to 30 minutes. Immediately on stopping the enzyme reaction, plates were read at 450 nm on a MultiSkan FC plate reader using SkanIt software. Sample concentrations were calculated using the published concentrations of the standards on a parametric logistic curve fit. Given the known loading concentration of total protein, the metabolite concentrations calculated by the plate reads were multiplied by the dilution factor for the specific sample, then multiplied by 1000 to give the concentration in ng metabolite per gram faeces as reported.

**Analysis of cytokines**

Point of kill blood was collected in vacutainer serum separation tubes and allowed to stand at room temperature for 1 hour as per the tube manufacturers guidelines. Samples were spun at 2000 g for 10 minutes in a refrigerated centrifuge and the serum supernatant transferred to a clean 1.5 ml Eppendorf. Serum was stored at -80°C until required.

Cytokine concentrations were determined using the Bio-Plex pro rat cytokine Th1/Th2 multiplex assay. This utilises pre coated magnetic beads to allow simultaneous detection of 12 cytokines in one sample. Cytokines targeted were GM-CSF, IFNγ, IL1a, IL1b, IL2, IL4, IL5, IL6, IL10, IL12 (70), IL13 and TNFα. The assay was run as per manufacturer’s instructions. In brief, serum samples were diluted 1: 4 in sample diluent and samples and standards were loaded in duplicate into wells preloaded with magnetic beads and incubated on a shaking plate at room temperature for 1 hour. Plate was washed using a BioPlex Pro wash station with magnetic plate to prevent bead loss. Detection antibodies were added to the plate and incubated on a shaking plate at room temperature for 30 minutes, protected from light by covering with aluminium foil. After another wash cycle, streptavidin-phycoerythrin was added and the plate incubated, protected from light, for a further 10 minutes at room temperature. The plate was washed again, and the beads were resuspended in assay buffer prior to reading. Plates were read on a BioPlex 100 system plate reader using its own software. The software used preloaded analyte detection parameters as set by the manufacturer. The standard curve created by the software for each plate was checked for outliers as recommended; however, no outlier removal was necessary. The observed concentration of each cytokine (expressed as pg/ml) as mapped to the standard curve was used for statistical analysis.

**3.1.6 Updated statistical analysis**

In a change from the Stage 1 report, all primary confirmatory statistical analysis were performed in Minitab v20. This change was due to the licence package available at the time and the experience of the researcher in using this software package. Calculations of within- and among-individual variability, and retrospective power analyses, were performed in R-studio as initially indicated in the F1000 Stage 1 report. Prior to analysis, we visualised and tested the data for the assumptions of the cognate statistical approaches (data distribution normality or heteroskedasticity), and when necessary, we used non-parametric alternatives or data transformation (square root transformation). A full description of the tests used by hypothesis is explained in sections 2.8.4 and 2.8.5. To account for potential Type I errors that may arise from multiple comparisons, we used Bonferroni correction where appropriate. We considered the p-value threshold of 0.05 as significant in our statistical comparisons unless stated otherwise.

**3.1.6.1 Power analysis**

Based on our previous data, the most variable response measure is total 50 kHz USVs with data taken from our own and other work ( LaFollette et al., 2018; Tivey et al., 2022) where treatments come closest to matching the proposed treatments. We have used these data to provide us with means per primary fixed effect combinations ( *i.e.*, treatment * sex) and pooled standard deviations, with resulting simulations based on the smallest effect size (0.09). Power analysis and sample size estimations were conducted using GLIMMPSE software. The desired power was set at 0.8, the Type I error rate at 0.05, the means scale factor was set at 1.0 and the variability scale factor at 1.3 using the Hotelling Lawley Trace test ( Hotelling, 1931). Simulations incorporated the non-independence of rats within a cage (cage fitted as a random effect), *via* a calculated intraclass correlation of 0.17 based on previous data ( LaFollette et al., 2018; Tivey et al., 2022). Additionally, repeated measures over six tickling treatment events/exposures were included in the power analysis. Our fixed effects were treatment (Control, P0, P1, P4), sex (male, female) and the treatment*sex interaction. Based on simulations detailed above, a sample size of n=16 rats ( *i.e.*, n=8 cages) per treatment and sex combinations, or n=64 total experimental units (128 total animals), provides a power of 0.829. In the results, we report retrospective effect sizes and uncertainty in our analyses.

**3.1.6.2 Blinding**

We were not able to blind the handler during the preparatory and experimental phases, as they needed to know which handling method they would be using with each rat. As individual rat’s behaviour was observed in the anticipation phase but not during the tickling sessions, we were able to blind the quantitative behavioural analysis as well as all other aspects including collection of USVs, analysis of physiological samples, and collation and analysis of behavioural data.

**3.1.6.3 Outlier extraction**

We are aware of the high variability in USV response among rats, and are experienced in recording it, and therefore did not exclude rats based on their USV production. For physiological markers, we used data points within the dynamic range of the assay and checked replicate quality. No outlier removal (based on the Grubb’s test ( Grubbs, 1969)) was performed. No animals were required to be excluded from the study for health reasons (e.g. injury or infection). One individual (female P1) displayed signs of sickness behaviour on the day they were scheduled to be killed. This cage was dispatched first to prevent prolonging any suffering. This individual was excluded from analysis of measures collected on the final day (prior to this final day, the animal fell within the parameters of its own group, suggesting there was no remarkable impact on earlier measures).

**3.1.6.4 Group level treatment, sex, day effects in USVs, physiological measures and behaviour**

For Objective 1 (Testing protocols varying in PH and pinning events against USV as a measure of affective state), mixed models were used with a restricted maximum likelihood (REML) estimation method with Kenward-Roger approximation for fixed effects. To test our Confirmatory Hypothesis (that indicators of positive affect are increased in treatments P0, P1 where PH is increased and amount of pinning reduced, relative to P4 and Control treatments), firstly sex and treatment were fitted as fixed effects, and cage and cohort were fitted as random effects. ^9^ No statistically significant effect of cohort or cage was observed on 50 kHz USV production. Two and three-way interactions were included in the model structure.

We found a significant sex*treatment interaction (F = 3.47, p = 0.018) Next, we included the factor time-point (day) in the model, which strengthened the fit and allowed the model to account for more of the variability in the data thus making it a stronger more robust model (without day R-sq adj = 38%, with day R-sq adj = 83%). The overall result is similar but the inclusion of day does allow the model to account for more of the variation in response (the more variation in response accounted for the more accurate the model).

For Objective 2 (Testing the behavioural and physiological effects of protocols varying in PH and pinning events) we used the same approach as described for Objective 1, and data were analysed in Minitab using Mixed Models with a restricted maximum likelihood (REML) estimation method with Kenward-Roger approximation to ascertain the effects of tickling treatments and sex on behavioural and physiological measures. For analysis of behavioural tests, sex and treatment were fitted as fixed effects and cage and cohort as random effects. Two-way interactions were included in the model structure. For the faecal corticosteroid analysis, sex, treatment and time point were fitted as fixed effects, with cage and cohort fitted as random effects. Two and three-way interactions were included in the model structure. To assess baseline effects of the treatments on FCM we have included analysis of timepoints 1 and 2. For cytokine analysis, mixed models (REML) were similarly used for those that met the model assumptions, with sex and treatment as fixed effects, and cage and cohort as random effects. Two-way interactions were included in the model structure. For those cytokines that did not meet model assumptions, Mann-Whitney U tests were performed on treatment alone, as there was no indication of sex differences being a factor in cytokine levels. No statistically significant effect of cohort or cage was observed on any of the outcome measures tested under objective 2. A full exploration of among- and within-individual variance by treatment was not possible as the data were too constrained for the model complexity, however a population level analysis to assess total variance in the model was performed.

**3.1.6.5 Exploratory analysis of within- and among-individual variability in USVs and FCM**

For Objective 1 to estimate the effect of treatment on both among-individual and within-individual variance in 50 kHz ultrasonic vocalizations (USVs), we employed a general linear mixed model ( Dingemanse & Dochtermann, 2013). To estimate the effect of treatment on the variance among individuals, we included a treatment specific effect for rat identity. To estimate the effect of treatment on the variance within individuals we included a treatment specific fixed effect to the residuals. To avoid inflating our estimates of variance components, the model also incorporated fixed effects for treatment, sex, and day, as well as random effects for cohort and cage. The repeatability of 50 kHz USV production was calculated using posterior estimates of among-individual and within-individual variances derived from the mixed model. These estimates of repeatability were adjusted for treatment, sex, and day effects, making them estimates of adjusted repeatability ( Nakagawa & Schielzeth, 2010). To evaluate whether the among-individual variance, within-individual variance, and repeatability of 50 kHz USV production varied across the different treatments, we compared the posterior distributions of these parameters between treatments. We report the posterior means and the 95% highest posterior density intervals (HPDIs) for these comparisons, providing a credible interval for each estimate. Variance components were estimated using the brms package v2.15 ( Bürkner, 2018) within RStudio 2021.09.1+. In brms, group effect estimates were provided as standard deviations which we converted into variances. Furthermore, as brms posterior residual variance is on the log-scale, we exponentiated these values. This approach allowed us to compare among-individual and within-individual variances on a consistent scale for calculating repeatability. The models for estimating variance components were run using four Markov Chain Monte Carlo (MCMC) chains, each with 8,500 iterations, including a burn-in of 1,000 iterations and a thinning interval of 100. We used uninformative or weak priors on all parameters ( Gelman et al., 2013) which included wide normal priors for fixed effects, and Half-Student priors for variance parameters.

### 3.2 Objective 1: Testing protocols varying in PH and pinning events against USV as a measure of affective state

**3.2.1 Confirmatory hypothesis 1**

We hypothesised that rats exposed to handling protocols that maximise playful interactions (i.e., where pinning number per session decreases) would show an overall increase in total 50 kHz USVs as an indicator of positive affect.

**3.2.1.1 Primary response variable: Number of USVs produced during treatment phase**

A primary analysis not including day as a factor indicated significant main effects of sex (F _1,113_ = 20.26, p < 0.001) and treatment (F _3,113_ = 19.49, p < 0.001) on mean 50-kHz USV production, and a significant sex*treatment interaction (F _3,120_ = 3.47, p = 0.018) on mean USVs produced. Overall females produced more USVs than males (T = 4.35, p < 0.001) and tickled animals produced more USVs than controls (p < 0.001) with the highest numbers of USVs being in the P1 groups (P1:169.79 vs P0:147.06 vs P4:145.61 vs C:79.113). The model fit was however found to be poor (R-sq = 37.7%) accounting for just over one third of the variability in response in the dataset, so an exploratory analysis including additional factors was performed to increase reliability.

**3.2.1.2 Exploratory hypothesis: Number of USVs produced during treatment phase using a model including the factor day**

To create a more accurate model, time-point (day) was included in the model as a fixed effect, improving model fit substantially (R-sq = 83.3%) allowing more reliable conclusions to be made. The previously observed main effects of sex (F _1,34.83_ = 20.02, p < 0.001) and treatment (F _3,32.72_ = 19.61, p < 0.001), and the sex * treatment interaction persisted (F _3,34.79 _= 3.72, p = 0.02) and additional two-way interactions of sex * day (F _5,599.05_ = 16.78, p < 0.001) and treatment * day (F _15,599.05_ = 2.42, p = 0.002) were observed. 22-kHz USVs were observed during early habituation for some individuals, however no 22-kHz USV were observed during the treatment phase.

**Figure 4.**
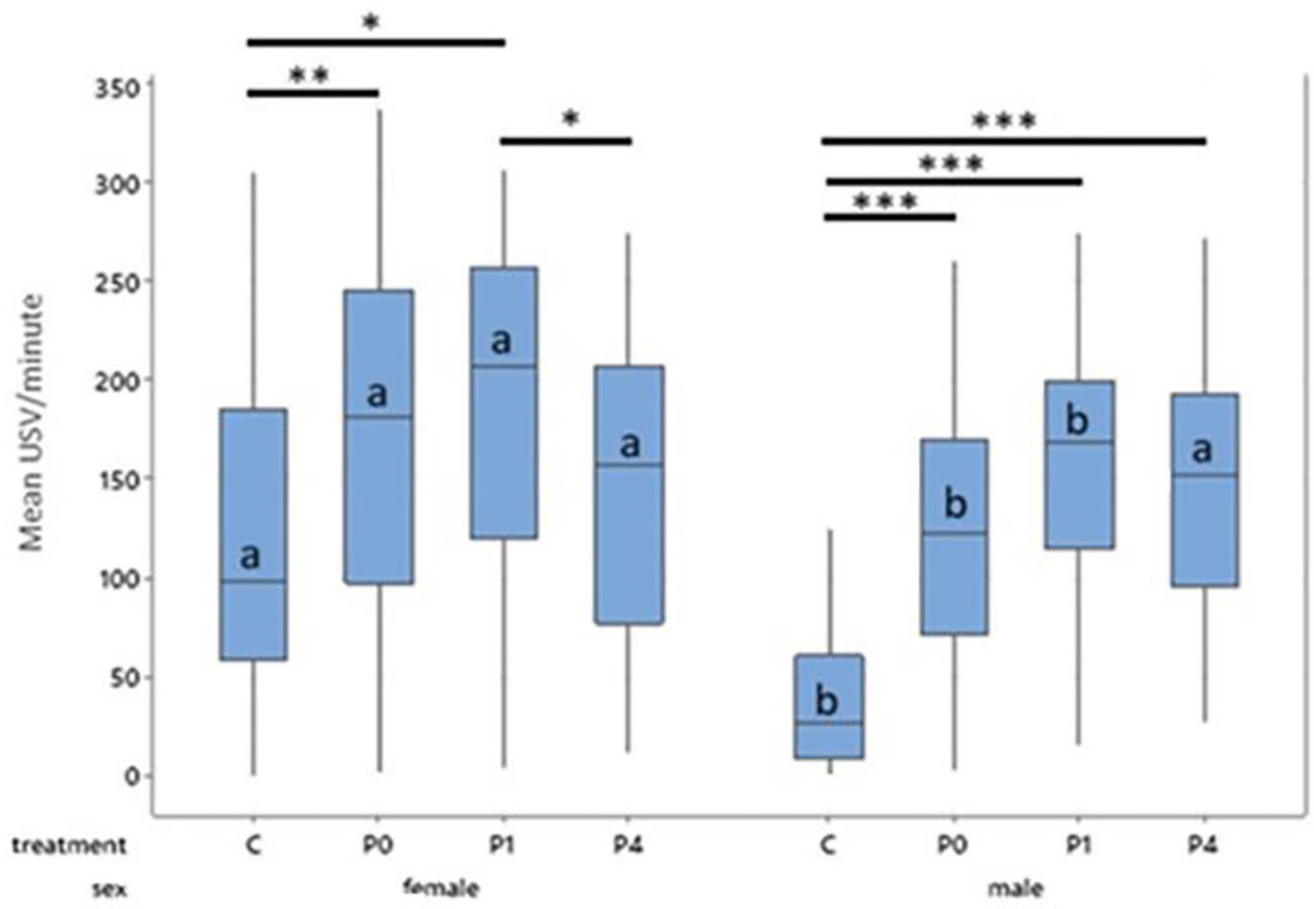
Mean 50 kHz USVs per minute during treatment for females and males by treatment across all days. Boxes represent interquartile range. Within sex, treatment differences are shown with * p < 0.05, ** p < 0.01 and *** p < 0.001. Between sex, columns of comparable treatments not sharing a letter are significantly different to one another at p < 0.05. For abbreviations see Figure 3.

As with the confirmatory analysis, females produced more treatment-induced 50 kHz USVs than males (155.2 vs 115.58, T = 4.47, p < 0.001) and tickled individuals produced more 50 kHz USVs than controls (p < 0.001), with the highest number of USVs being produced by the P1 group. Females in the P1 group produced significantly more 50 kHz USVs than those in the P4 (T = 2.19, p = 0.03) and control groups (T = 3.67, p = 0.016), but not more than those in the P0 group. Female P0 rats produced significantly more 50 kHz USVs than female control rats (T = 2.96, p = 0.009). Female rats on P4 or control did not differ in total 50 kHz USV production. In males, controls produced fewer 50 kHz USVs than P0 (T = -4.71, p = 0.001), P1 (T = -6.59, p < 0.001) and P4 males (T = -6.05, p <0.001; Figure 4). Male P0, P1, and P4 rats did not differ significantly from each other.

Female controls, P0 and P1 all produced more 50 kHz USVs during treatment than their male counterparts within the same treatment (Control, t = 9.14, p < 0.001; P0, t = 5.57, p < 0.001; P1, t = 3.27, p = 0.024). Males and females in the P4 group did not differ in their 50 kHz USV production ( Figure 4). Male control rats produced fewer 50 kHz USVs than all other groups both between and within sex (p < 0.001).

**Figure 5.**
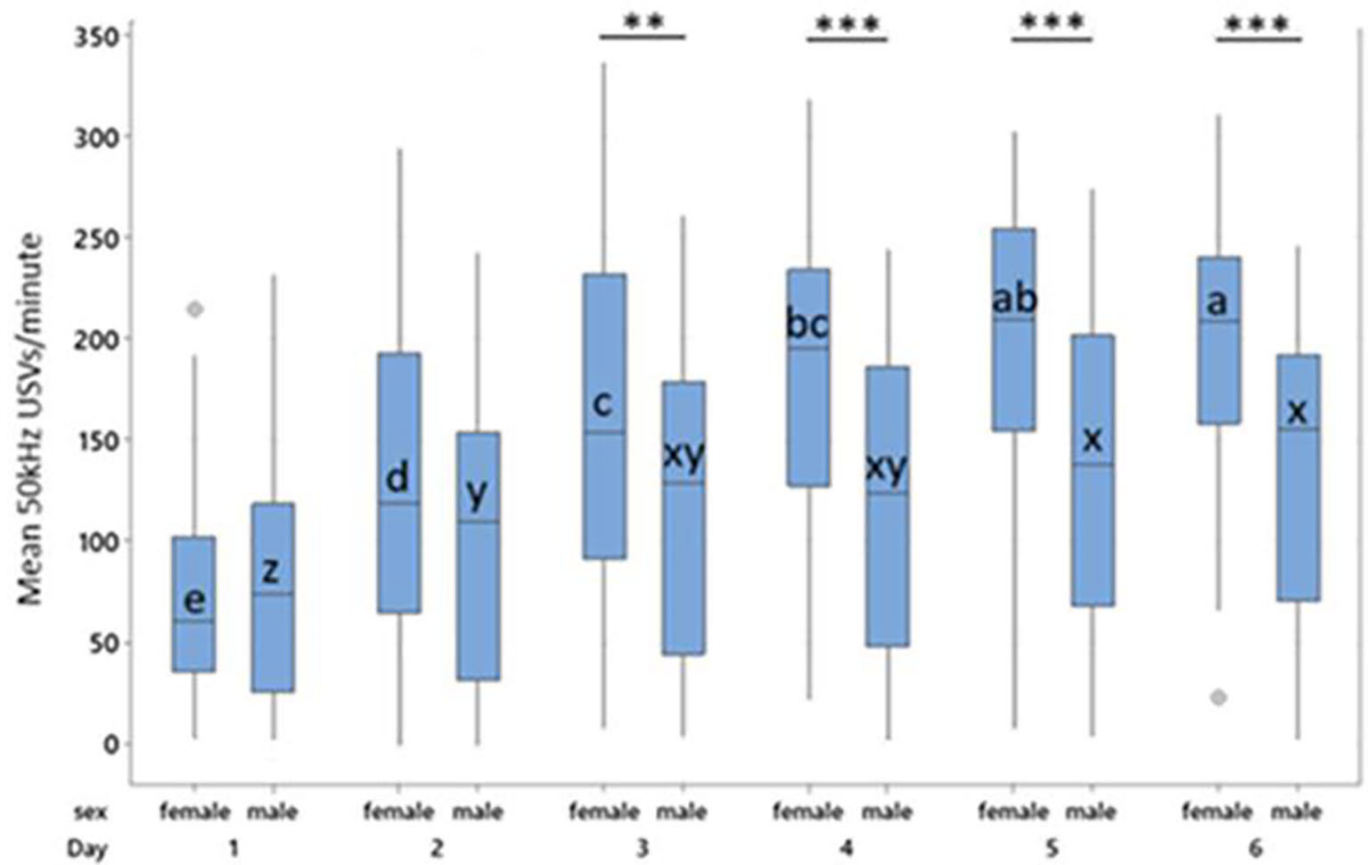
Mean 50 kHz USVs per minute during treatment between sex per day across all treatment groups. Boxes represent interquartile ranges, outliers are represented by a grey dot. Between sex, within day pairwise differences are shown with **p < 0.01 and ***p < 0.001. Within sex, columns that do not share letters are significantly different to one another at p < 0.05.

50 kHz USV production increased over the treatment phase in a dose dependent manner that differed by sex ( Figure 5). 50 kHz USV production continued to rise in females to Day 5, while males reached a plateau at Day 3. On Days 5 and 6, female rats did not differ and produced more 50 kHz USVs than all days and groups bar female rats on Day 4. Males and females did not differ in their 50 kHz USV production on Days 1 (T = 0.44, p = 1.00) and 2 (T = 2.35, p = 0.45; Figure 5).

**3.2.2 Exploratory hypothesis: Analysis of within and between individual variation** ^10^

We predicted that 50 kHz USV measures would be less variable among individuals in treatments with less pinning.

**3.2.2.1 Primary response variable: USV variability within and among individuals**

Across all four treatments, rats exhibited among-individual variance in 50 kHz USVs, indicating variability in total vocalizations between different individuals ( Table 2). Comparisons between treatments did not reveal significant differences in among-individual variance, as all 95 % credible intervals for the differences included zero.

Within-individual variance in USVs was present across all play treatments, reflecting variability in vocalizations within the same individual over time ( Table 2). Notably, within-individual variance was higher in the P0 treatment compared to the Control (mean difference = 0.105, 95 % CI 0.017-0.195) and P4 (mean difference = 0.123, 95 % CI 0.039-0.209) treatments, indicating that the USVs of individuals fluctuated more over time in the P0 group. Similarly, rats in the P1 treatment also exhibited more fluctuations in USVs over time compared to the P4 (mean difference = 0.073, 95 % CI 0.002-0.138) treatment, although the 2.5 % credible interval is close to zero, indicating a marginal difference. USVs were consistently repeatable across treatments, as indicated by the adjusted repeatability estimates ( Table 3). However, there were no differences in repeatability between treatments, as the credible intervals for repeatability differences included zero.

**Table 3.** Posterior estimates for the treatment effects on variance among individuals, variance within individuals, and adjusted repeatability of rat USVs. Mean estimates are displayed alongside their 95% credible intervals (CI). Variance is considered significant (indicated in bold) where the credible intervals quoted do not include 0.

	Model estimate	95 % Credible Interval	
	Mean	2.5 %	97.5 %
Variance among individuals			
Control	**0.320**	0.126	0.545
P0	**0.506**	0.219	0.821
P1	**0.374**	0.185	0.618
P4	**0.407**	0.188	0.664
Variance within individuals			
Control	**0.219**	0.172	0.271
P0	**0.324**	0.260	0.404
P1	**0.272**	0.218	0.337
P4	**0.200**	0.159	0.247
Adjusted Repeatability			
Control	**0.578**	0.397	0.741
P0	**0.597**	0.440	0.749
P1	**0.567**	0.413	0.718
P4	**0.657**	0.516	0.804

**3.2.3 Secondary response variable: Waveform parameters**

The delta frequency of the 50 kHz USVs produced differed by treatment (F _3,120_ = 10.90, p < 0.001), day (F _2,240_ = 53.35, p < 0.001) and sex (F _1,120_ = 10.54, p = 0.002). Variance components indicate individual contributes to 39.1 % of the total variance (Z = 4.96, p < 0.001). Overall, the delta frequency was lower in the control individuals than rats in the other treatment groups (P0: T = 4.13, P < 0.001; P1: T = 5.46, p < 0.001; P4: T = 3.61, p < 0.001). Delta frequency increased over days, with significant differences observed between Days 1 and 3 (T = 6.68, p < 0.001) and Days 3 and 6 (T = 3.49, p = 0.002). Delta frequency was overall higher in females than males (T = 3.25, p = 0.002).

The sinuosity of the 50 kHz USVs produced did not differ by sex, but did differ by treatment (X ^2^ = 12.08, df = 3, p = 0.007) and day (X ^2^ = 8.69, df = 3, p = 0.013). Differences determined by non-overlapping confidence intervals indicate sinuosity to be higher in P1 and P0 than in P4 and control and at Day 6 compared to Day 1.

**3.2.4 Secondary response variable: Number of USVs produced during anticipation**

Differences in 50 kHz USV production during the anticipation phase were observed in a two-way sex * day interaction (F _5,599.07_ = 13.13, p < 0.001), but were not influenced by treatment. No 22-kHz USVs were observed during the anticipation phase.

**Figure 6.**
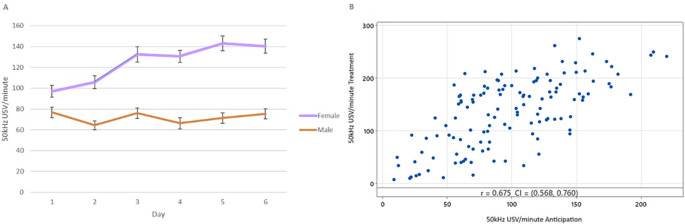
Panel A - 50 kHz USVs/ minute during the anticipatory phase for female (purple) and male (orange) for each day. Error bars shown are standard error of the mean. Panel B – distribution of mean 50 kHz USV/minute during the treatment phase plotted against mean 50 kHz USV/minute during the anticipation phase for each rat (N = 128).

Females produced more anticipatory 50 kHz USVs than males (124.9 vs 71.67 T = 8.31, p < 0.001). Female anticipatory USVs increased over time, while male anticipatory USVs did not ( Figure 6 panel A). A moderate correlation (r ^2^ = 0.675, p < 0.001) was observed between mean anticipatory 50 kHz USVs and 50 kHz USVs during treatment ( Figure 6 panel B).

**3.2.5 Secondary response variable: Behaviour during anticipation**

**Figure 7.**
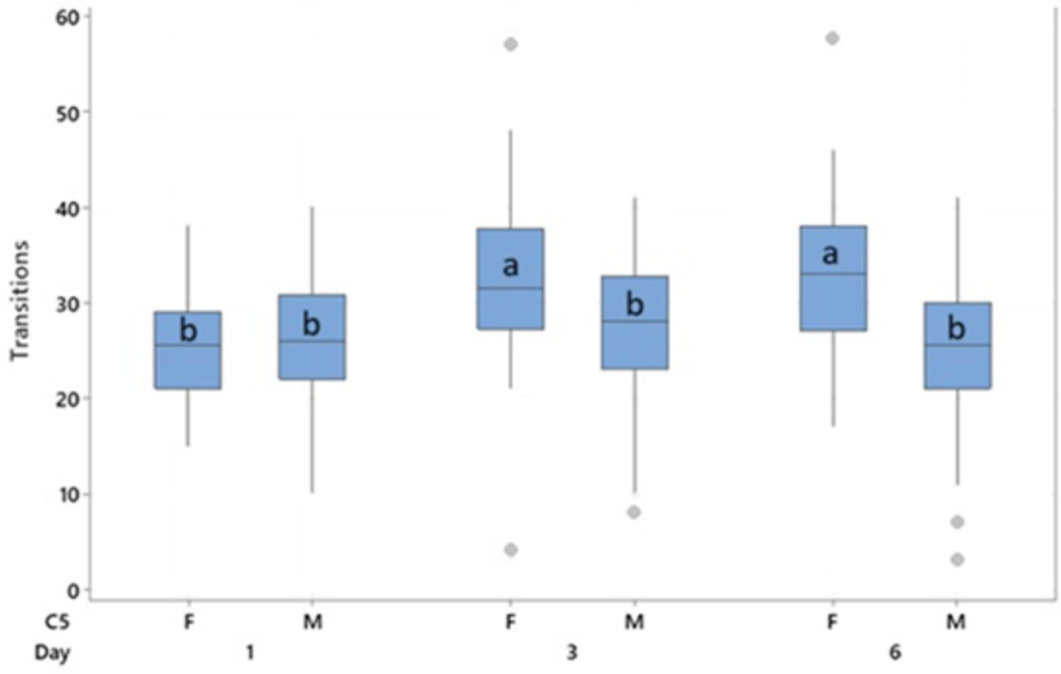
Mean behavioural transitions during the anticipatory phase by day. Boxes represent interquartile range, outliers are represented by a dot. Bars indicated by “a” are significantly different to all others by pairwise comparison with p < 0.001. Bars which share a letter do not differ from each other.

Behavioural transitions during the anticipation phase were measured on days 1, 3 and 6 and found to differ with a sex * day interaction (F _2,240_= 19.18, p < 0.001). No effects of treatment were observed. Females performed more behavioural transitions than males (30.3 vs 26.1, T = 4.54, p < 0.001). Transitions were lower on day 1 than on days 3 (T = -6.02, p < 0.001) and 6 (T = -4.75, p < 0.001), primarily driven by the increase in transitions observed in females on days 3 and 6 through the interaction of sex * day ( Figure 7). Male transitions did not increase over time.

A weak (r ^2^ = 0.337, p < 0.001) positive correlation was observed between the number of behavioural transitions and anticipatory 50 kHz USVs produced. The expression of social play in the five minutes prior to the anticipatory phase was too infrequent for analysis.

**3.2.6 Effect size**

Retrospective power analysis using the variability within this data set to calculate effect of Treatment x Sex X Day (Between x Between X Within), with an intraclass correlation coefficient by cage of 0.42, returned a power level value of 0.8697, exceeding our predicted value of 0.80.

### 3.3 Objective 2: Testing the behavioural and physiological effects of protocols varying in PH and pinning events

Exploratory hypothesis: We hypothesised that the behavioural and physiological consequences of tickling would be less variable among individuals and more repeatable when rats were exposed to handling protocols that maximise playful interactions and where pinning episode number per session decreases.

**3.3.1 Primary response variables: Elevated plus maze (EPM) and open field (OF)**

There was a significant effect of sex (F _1,113_= 11.34, p = 0.001) and treatment (F _3,113_= 6.61, p < 0.001) on total distance travelled in the EPM. Females travelled further than males (T = 3.37, p = 0.001) and treatment group P1 travelled a shorter distance than all other groups (Control: T = -3.09, p = 0.013; P0: T = -3.20, p = 0.009; P4: T = -4.20, p < 0.001). Percent time in and % distance travelled in the open arm were not related to fixed factors in the model. No correlations were observed between any of the measures in the EPM and USV production on the final day of testing.

Sex effects were observed in total distance travelled (F _1,52.78_= 8.47, p = 0.005), % distance travelled in outer zone (F _1,53.34_= 10.78, p = 0.002) and % time in outer zone (F _1,51.75_= 35.26, p < 0.001) in the open field test. Females travelled further than males overall (T = 2.91, p = 0.005). Males spent a greater percentage of time in the outer zone (T = 5.94, p < 0.001) and a greater percentage of their distance travelled was in the outer zone (T = 3.38, p = 0.002) compared to females.

Weak negative correlations were observed between USV production on the final day of treatment and % distance travelled in the outer zone (r ^2^ = -0.334) and % time in outer zone (r ^2^ = -0.362). No treatment effects were observed in the open field test. There was no correlation between post-test physiological measures (interleukins, corticosterone metabolites) and test outcomes (total distance in either EPM or OF, and respectively time in open arm and time in the centre).

**3.3.2 Secondary response variables: physiological and inflammation related variables**

**3.3.2.1 Faecal corticosteroid metabolites (FCM)**

Differences were observed in concentration of FCM with a sex * timepoint (F _1,238.03_= 9.05, p < 0.003) interaction. No effects of treatment were observed.

**Figure 8.**
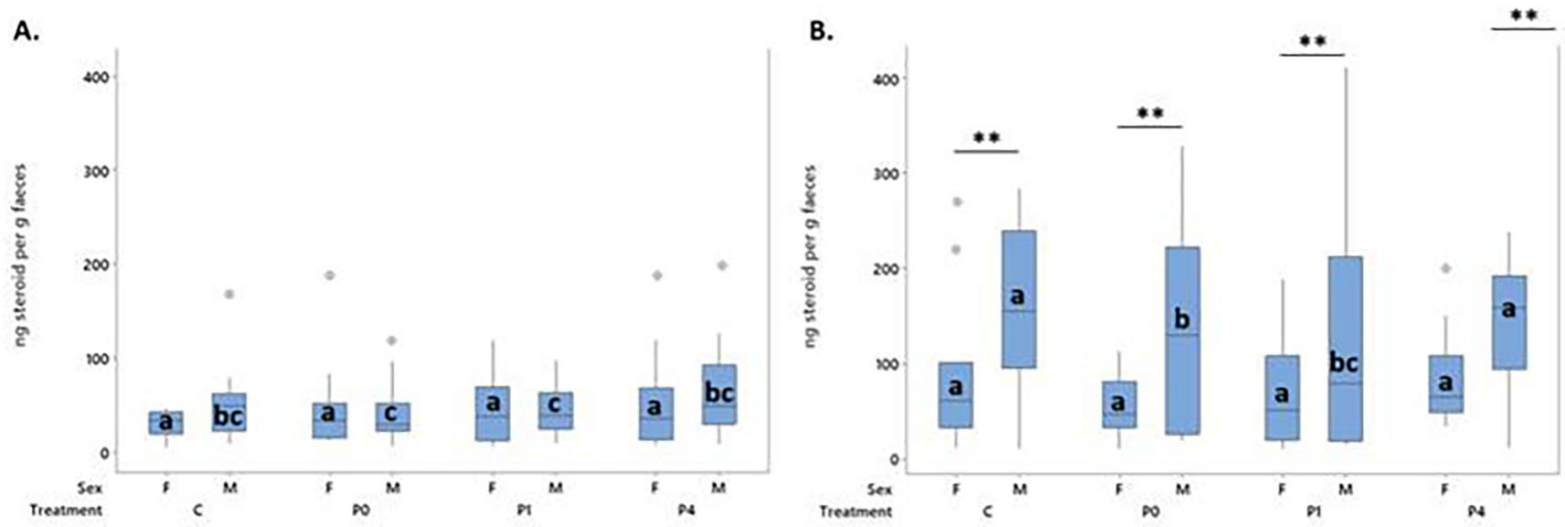
Faecal corticosteroid metabolite concentrations prior to experimental phase (TP1 – Panel A) and end of experimental phase (TP2 – Panel B). Males and females did not differ at TP1. Between sex (within time point) differences were only observed across treatments at TP2 (Panel B). Within sex (across timepoints), columns that do not share letters are significantly different to one another at p < 0.05.

Overall, males excreted higher levels of FCM than females (T = 4.52 p < 0.001; Figure 8, Panel B), and concentrations at timepoints 2 (at end of experimental phase; TP2) were higher than at time point 1 (prior to experimental phase; TP1) (TP1 vs TP2 T = 7.77, p < 0.001). At TP1, no differences were found between sexes or allocated treatment groups ( Figure 8, Panel A). The % change in FCM over the treatment phase (TP1 to TP2) differed significantly by sex (F _1,113_= 6.54, p = 0.012) but not treatment, with males increasing on average 2.5 x more than females (mean % change 464.4 vs 199.4 respectively).

A weak, negative correlation (r = -0.336) was observed between USV production on the final day of treatment and FCM at the end of the treatment phase.

**3.3.2.2 Faecal corticosteroid metabolite variability within and between individuals**

Across all four treatments, rats did not exhibit among-individual variance in FCM, indicating that the average amount of FCM excreted did not vary across individuals in any treatment groups ( Table 3). Comparisons between treatments did not reveal significant differences in among-individual variance, as all 95 % credible intervals for the differences included zero.

Within-individual variance in FCM was observed across all treatments, reflecting fluctuations in the amount of FCM excreted within the same individual over time ( Table 4). Within-individual variance was greater for rats in the Control treatment compared to the P4 treatment (mean difference = 0.134, 95 % CI 0.001-0.287).

**Table 4.** Posterior estimates for the treatment effects on variance among individuals, variance within individuals, and adjusted repeatability of rat FCM. Mean estimates are displayed alongside their 95% credible intervals (CI).

	Model estimate	95 % Credible Interval	
	Mean	2.5 %	97.5 %
Variance among individuals			
Control	0.020	0.000	0.076
P0	0.016	0.000	0.061
P1	0.028	0.000	0.106
P4	0.019	0.000	0.068
Variance within individuals			
Control	**0.390**	0.265	0.541
P0	**0.351**	0.232	0.479
P1	**0.409**	0.277	0.579
P4	**0.261**	0.175	0.365
Adjusted Repeatability			
Control	0.047	0.000	0.170
P0	0.042	0.000	0.149
P1	0.061	0.000	0.220
P4	0.065	0.000	0.225

Across all four treatments we did not find that FCM was repeatable ( Table 4) and there was no difference in repeatability across treatments. The absence of repeatability was driven by the low amount of among-individual variance combined with high within-individual variance across all treatment groups.

**3.3.2.3 Cytokine multiplex assays**

Statistically significant effects of treatment were observed for serum concentration of IL5 (x ^2^ = 16.05, df = 3, p = 0.001), IL13 (x ^2^ = 13.55, df = 3, p = 0.004), IL10 (F _1,126_ = 4.52, p = 0.005) and IL12 (F _1,126_ = 3.09, p = 0.03). Group wise differences in IL5 and IL13 are reported using non-overlapping confidence intervals as post-hoc tests are not compatible with Mann-Whitney analysis. IL10 and IL12 were analysed by REML and pairwise Tukey tests reported. For IL5, the order of median concentrations was Control < P1 < P4 < P0 with the differences derived from non-overlapping confidence intervals occurring between control and P4 individuals. For IL13, the order of median concentrations was Control < P1 < P0 < P4 with the differences derived from non-overlapping confidence intervals occurring between control and P0 individuals. For IL 10, the order of mean concentrations was Control < P1< P4 < P0, with the differences derived from post-hoc comparisons occurring between Control and P4 (T = 2.82, p = 0.028), and Control and P0 (T = 3.23, p = 0.009). For IL12, the order of mean concentrations was Control < P1 < P4 < P0, with the differences derived from post-hoc comparisons occurring between control and P0 (T = 2.76, p = 0.034). No significant effects of sex or treatment were observed for IL1a, IL1b, IL4, IL6, IL2, GM-CSF, TNF or IFN. No correlations were observed between any cytokines tested and faecal corticosteroid metabolites, or total USV production.

**3.3.2.4 Bodyweight**

Growth, as measured by % increase in body weight, was not affected by treatment (F _3,116_ = 0.02, p = 0.996). Sex effects in % change in body weight were observed (F _1,116_ = 39.29, p < 0.001) with males increasing their body weight more than females (Male mean 174.4 g, SE 2.43: Female mean 153.8 g, SE 2.81).

## 4. Discussion

For Objective 1, our Confirmatory Hypothesis 1 was that juvenile rats exposed to playful handing (PH; tickling where pinning is minimised in favour of more flexible interactions between the hand and rat), would show an overall increase in total 50 kHz USVs as an indicator of positive affect. Our results are generally in agreement with this hypothesis which is based on our earlier opinion paper ( Bombail et al., 2021), where we hypothesised that pinning was responsible for individual variation in response to heterospecific play. We show here a significant effect of tickling with reduced pinning on total 50 kHz USVs with treatment P1 producing significantly more USVs than other treatments; treatments P0 and P4 were not different to each other but different from controls. However, a significant sex by treatment interaction indicates that it was females who were responsible for this effect. Within sex comparisons reveal that females in the P1 treatment produced the highest levels of USVs at statistically different levels to female P4 and controls. P0 females produced statistically similar levels of USVs to female P1; female P0 levels were not different to P4 but were different to controls. P4 females did not differ from controls. In contrast, none of the male rats on different handling treatments differed from each other and their rates of USVs were significantly higher than those of control males.

These results suggest that females prefer tickling where pinning is minimised, whereas males do not discriminate across treatments varying in amount of pinning, on the basis of USV production, a well-validated indicator of positive affective states in rats ( Burgdorf & Panksepp, 2001). This is a novel finding as there has been no previous direct comparison of sex differences in response to tickling treatments involving different amounts of pinning. One explanation for this sex by treatment interaction is that it reflects sex differences in social play behaviour. Studies of social play in juvenile rats have revealed sex differences in playful defence behaviours, with males more likely to adopt a supine posture and females more likely to evade through lateral rotation ( Pellis & McKenna, 1992; Pellis & Pellis, 1990). Thus, forceable pinning, as applied in the standard approach to rat tickling, could be antagonistic to females’ evolved preferences for playful defence. Even when playing with males and being exposed to more nape attacks, females were more likely to evade with lateral rotation ( Pellis & Pellis, 1990); one explanation of this being females’ greater capacities to sense the approach of an attacker ( Pellis et al., 1994) and hence being more able to perform longer distance defence moves ( Pellis et al., 1989, 1997). It is worth noting that this finding might need to be taken with caution in the light of strain ( Himmler et al., 2014a and 2014b) and age ( Pellis et al., 2006) differences in the occurrence of pin behaviour during play.

Between-sex comparisons show that females in P1, P0 and control groups produced significantly more USVs compared to males in the same treatments; female and male P4 rats did not differ in their USV production. This suggests that except for the P4 treatment, females found the treatments applied here including the control conditions more rewarding than males. Evidence from secondary response variables (USVs and behavioural transitions observed during the one-minute anticipatory phase) supports the interpretation that females were more positively anticipating their allotted treatment than males. Tivey et al., (2022) also found that after 10 days of playful handling, females produced more USVs than males with an indication that female controls also increased calling relative to male controls. The increased level of USV response to playful handling is opposite to what would be predicted from social play where male juvenile rats are reported to find social play more rewarding than females. Juvenile male rats exhibit more playful initiation behaviour than females ( Meaney & Stewart, 1981; Pellis & Pellis, 1990; Thor & Holloway, 1984) and produce more USVs than females during social play including during pinning ( Kisko et al., 2021).

However, direct comparisons between social play and tickling to interpret USV production need to be done with caution, as these are potentially not equivalent experiences for the rat. For example, the patterns and correlations between USVs and specific behaviours are known to vary across contexts ( Burke et al., 2022; Melotti et al., 2014). In male rats the strongest associations between USVs and behaviour in social play are between approaching, following and nape contact whereas with tickling the strongest association is with pinning ( Burke et al., 2022; they applied the standard tickling protocol). Burke et al. (2022) suggested that for male rats being pinned during tickling may be important for production of USVs in comparison to social play where de-vocalisation studies show that it is typically the rat doing the pinning that emits these calls ( Burke et al., 2018). Comparable data for females are not currently available.

Another distinctive difference between social play and tickling in the Burke et al., (2022) study were the correlations between behaviours and sub-types of USVs. Burke et al. (2022) report that in social play, the strongest associations between behaviour and USVs involve frequency modulated (trill) calls, whereas in tickling the strongest associations involve flat calls. Burke et al. (2022) propose that the lack of correlation between trills and specific tickling behaviour indicates that trills are produced relatively indiscriminately during tickling, potentially due to the lack of a conspecific play partner. Variations in call sub-types could be crucial when interpreting rats’ affective response to different forms of play. Trill calls are seen to be most clearly associated with positive affect ( Burgdorf et al., 2008) whereas flat calls are thought to be more involved in social communication ( Wöhr et al., 2008). There are also known to be sex differences in call sub-types during playful handling. Tivey et al., (2022) found that playfully handled and control females produced more flat calls associated with play behaviours, performed during the playful handling sessions, than did males; in contrast both sexes produced trills in response to playful handling. We did not analyse sub-types of USVs but report here both treatment, sex and day effects on the waveform parameters derived from DeepSqueak. Although there is currently no direct validation of these parameters against manually scored call sub-types, we could expect lower delta frequencies and lower sinuosity scores to reflect flat calls and higher delta frequency and higher sinuosity scores to reflect trill calls. In line with this, the lower delta frequency in the control groups and the increasing delta frequency over days in the treatment groups, could suggest increases in trill calls with exposure to heterospecific play. The higher delta frequency in females could reflect their overall higher trill call rate in response to control and treatment conditions. The data on sinuosity could also be consistent with higher levels of trill calls in the treatments with reduced levels of pinning.

Our Confirmatory Hypothesis 2 was that 50 kHz USVs would be less variable in treatments with less pinning. Generally, the literature suggests that individual rats vary consistently in their USV production although most studies use adult male rats (e.g., Ahrens et al., 2013; Sundarakrishnan & Clarke, 2022). Given that we had multiple USV measures for rats over time we were able to explore individual variation among and between them. We also reported repeatability statistics which quantify the proportion of total USV production that can be attributed to differences amongst individuals relative to total phenotypic variance. To our knowledge, this is the first study that partitions among- and between-individual variation in USVs in a single model. The results do not support our hypothesis as among-individual variation did not differ between treatments and within-individual variation was less consistent in treatments with less pinning.

For Objective 2 our Exploratory Hypothesis was that behavioural and physiological consequences would be less variable in treatments that minimise pinning. Results from our primary response variables (behavioural responses to the elevated plus maze (EPM) and open field test (OFT)) carried out at the end of the treatment phase, do not provide strong support for this hypothesis. The treatment and sex effects on distance travelled in the EPM were not matched by other parameters such as time in the open arms of the maze. We found no treatment effects on OFT parameters. The sex effects observed in the OFT were indicative of lower anxiety in females, and this is in agreement with recent findings on sex differences in the OFT ( Knight et al., 2021).

Our secondary response variables for Objective 2 were physiological parameters which we anticipated would reflect the positive affective responses to reducing pinning. The results on faecal corticosteroid metabolites (FCM) do not support our hypotheses as changes in FCM over the treatment phase (timepoints 1 and 2) indicate effects of sex rather than treatment. Sex differences in FCM in rodents are already relatively well established ( Cavigelli et al., 2005). Our variance analysis did find that treatment P4 was associated with the lowest and P1 with the highest within-individual variance, but the analysis did not find significant differences in among-individual variance across treatments. Overall, our analysis reveals a lack of repeatability in FCM.

We also measured serum concentrations of a panel of cytokines anticipating that treatments with less pinning would be reflected in changes reflective of an anti-inflammatory profile. Our results do not support our hypothesis. We found treatment effects on 4 out of 12 cytokines, with control animals having consistently the highest concentrations followed by P1 with P0 and P4 alternating with having the lowest concentrations. Of the 4 cytokines with treatment effects, IL5 and IL12 are classed as pro-inflammatory ( Pelaia et al., 2019; Trinchieri, 1995) and IL10 and IL13 as anti-inflammatory ( Iyer & Cheng, 2012; Kolosowska et al., 2019).

### 4.1 Summary and implications

We report here on a scientifically based refinement of tickling protocols in male and female juvenile rats. We tested the hypothesis that individual variation in affective response to tickling would be reduced with less pinning (where the rat is placed on their back and vigorously tickled on their ventral surface). We used total USV production as our main outcome measure of rats’ affective response to tickling. We also explored the hypothesis that tickling with reduced pinning would lead to less variation among individuals in other behavioural and physiological measures.

Our results confirm that tickling reliably induces increased USV production indicating a positive effect on rats’ affective state, in both male and female rats. We also report a sex difference in response to pinning with females preferring tickling with one pin per session. Although in males the tickling treatments did not differ significantly, the one pin per session treatment was the numerically highest treatment and significantly different from controls in terms of USVs. Thus, harmonising to one pin per session for both sexes would be consistent with our findings and help with implementation across facilities. Levels of USVs indicate that females generally found treatments including control conditions more rewarding than did males. We were not able to confirm our hypothesis that tickling with less pinning would lead to less variation among individuals in USV production and in other behavioural and physiological measures.

These results indicate the need to consider sex differences when developing welfare interventions. Our work provides the basis for a refined tickling protocol that can be used to improve the welfare of both sexes of rats in a practically feasible way.

Future work could test our protocol on rats of different strains and ages and provide further validation using behavioural tests of affective state.

### 4.2 Study status

This study has now been completed.

### 4.3 Study limitations

We assigned animals to treatments according to preset criteria to homogenise factors we assume might affect treatment response (sex, litter of origin and social cage environment, body weight). Our pseudo-randomisation procedure is therefore more deterministic than the computer-run randomisation algorithm. Additionally, the experimenter was aware of the treatments administered as the rats were handled. He was therefore not blinded to the treatments. The effect of this was limited by the lack of discernible patterns (order of passage pseudo-randomisation) and impossibility to memorise treatments for each of 64 cages at the time of analysis. A different researcher, who had not handled the rats and was blinded to their treatment allocation, performed the faecal corticosteroid metabolite assays and also performed the statistical analysis thus reducing potential biases in analysis. Finally, we accept that use of a single male handler constitutes a limitation, especially in the light of recent reports about experimenter sex affecting rodents.

## Data Availability

Figshare: Data for stage 2 registered report, https://doi.org/10.6084/m9.figshare.28248773.v1 ( Bombail, 2025a). This project contains the following underlying data: AllData For Repository.xlsx Data are available under the terms of the Creative Commons Attribution 4.0 International license (CC-BY 4.0). ARRIVE guidelines for ‘Stage 1 Registered Report: Refinement of tickling protocols to improve positive animal welfare in laboratory rats’: https://doi.org/10.6084/m9.figshare.20914993 ( Bombail, 2022). Data are available under the terms of the CC0 license. ARRIVE guidelines for ‘Stage 2 Registered Report: Refinement of tickling protocols to improve positive animal welfare in laboratory rats’: https://doi.org/10.6084/m9.figshare.28248698.v1 ( Bombail, 2025b) Data are available under the terms of the Creative Commons Attribution 4.0 International license (CC-BY 4.0).

## Footnotes

^1^
In line with the Registered Report process, we cannot deviate from the introduction of the original submission; in section 3.1.1 we describe clarifications on further analyses we carried out to investigate individual variability.

^2^
As required in Stage 2 reporting, this section contains the original planned methodology, at the time of Stage 1 publication. Since then, a number of alterations have taken place, please refer to section 3.1.

^3^
Please refer to sections 3.1.3 and 3.1.5 for details of alterations to Data Collection since the Stage 1 publication.

^4^
This was performed on single rats in the tickling arena, please see section 3.1.3 for amendments to the original protocol.

^5^
22kHz USV were too rare to analyse.

^6^
As required in Stage 2 reporting, this section contains the original planned methodology, at the time of Stage 1 publication. Further details on the EPM and OF protocols are described in section 3.1.2.

^7^
Steroid and cytokine assays were performed according to different protocols to those described in the Stage 1 paper. As required in Stage 2 reporting, this section contains the original planned methodology, at the time of Stage 1 publication. The alterations to the protocols are described in section 3.1.5.

^8^
This section contains the original planned methodology, at the time of Stage 1 publication. Alterations are described in section 3.1.6.

^9^
In the Stage 1 paper we mistakenly omitted cohort as random effect, also as an exploratory analysis the factor time-point (day) was later added to generate a more robust statistical model (see 3.2.1.2).

^10^
As we chose a different (improved) approach to the Stage 1 paper for estimating individual differences in USVs we report these results as Exploratory (see Section 3.1.6.5 for details of the approach).

^11^
The ethogram was modified post Stage 1 report to observing single animals during the anticipation phase in the tickling arena, instead of pairs as was originally planned, please see section 3.1.4 for further details.
