# Supplementary material for: Stage 2 Registered Report: Refinement of tickling protocols to improve positive animal welfare in laboratory rats
Source: F1000Res. 2025 Sep 23;14:773. Originally published 2025 Aug 6. [Version 2] doi: 10.12688/f1000research.166417.2 (PMC12438952; doi:10.12688/f1000research.166417.2)
Supplement: Peer review history

# Reviewer report: Reviewer response for version 2

**DOI:** 10.5256/f1000research.188431.r416643

**Published:** 2025-09-24

**Authors:** Sergio Pellis

**Affiliations:** 1 University of Lethbridge, Lethbridge, Canada

I have no further concerns.

Is the work clearly and accurately presented and does it cite the current literature?

Yes

If applicable, is the statistical analysis and its interpretation appropriate?

Yes

Are all the source data underlying the results available to ensure full reproducibility?

Yes

Is the study design appropriate and is the work technically sound?

Yes

Are the conclusions drawn adequately supported by the results?

Yes

Are sufficient details of methods and analysis provided to allow replication by others?

Yes

Reviewer Expertise:

Behavioural neuroscience, animal behaviour

I confirm that I have read this submission and believe that I have an appropriate level of expertise to confirm that it is of an acceptable scientific standard.

# Reviewer report: Reviewer response for version 1

**DOI:** 10.5256/f1000research.183401.r404440

**Published:** 2025-09-15

**Authors:** Carla Forte Maiolino Molento

**Affiliations:** 1 Federal University of Paraná, Curitiba, Paraná, Brazil

General comments:

 This is a relevant study with practical consequences which are impactful for millions of rats kept in laboratories worldwide. The results suggesting that females prefer tickling where pinning is minimised, whereas males do not discriminate across treatments varying in amount of pinning, allow for an important refinement of recommended practices, which can be then promoted to provide for increased welfare status for the rats. Hopefully the findings will be disseminated and incorporated in bioteries and laboratories, thus directly touching the animals. This is the priority feedback I’d like to leave from reading the report.

 As a form of contribution, I will leave some suggestions, for the consideration of the authors.

 Is ‘heterospecific play between rats and the human hand’ the best way to describe the interaction? How can it be with only the hand? This is a much broader interaction which, from my point of view, tends to affect both subjects participating in it. I suggest ‘heterospecific play between rats and humans’

 Results regarding the significance of the effect of day suggest that you are following the process of establishing a tickling routine, rather than observing the plateau of affective consequences when a routine is in place. Perhaps analyzing only the last session could give a hint on what the situation will look like for an established situation. It would be interesting to test what the conclusions would be in this case.

 It is surprising that anticipatory vocalizations and behaviours did not differ across treatments. Does this mean that the simple attention given, by moving the animals from their flat routine, has welfare benefits? How did you test anticipatory results in themselves, wouldn’t knowledge regarding basal behaviours, i.e. those in other moments of the day, be required? In other words, how can results be differentiated from overall behaviours of the individuals, unrelated to the expectation of any upcoming event – in our case, experimental events of each treatment?

 Do the additional physiological measures obtained through killing of the animals justify this decision? This is important to discuss, as it impacts future decisions. Were animals killed by the same person who performed the experimental treatments? Please add a paragraph on this, discussing how the researcher and the person performing the procedures, felt regarding killing the rats.

 Overall, the discussion section felt disproportional to the rest of the report, less detailed and shorter than expected.

 As a logistic comment on the reviewing activities, it was different to act as a reviewer in the report style presented, as compared to the more common scientific paper review. It would be more efficient to work with a more advanced scientific style, in a paper format, as this is the most relevant product for the advancement of knowledge and presents a more mature and concise writing. In addition, I was in doubt on whether it was expected that I evaluate the changes in the initial research proposal – which I have not done, as it would require the availability of previous versions. It will be beneficial to make it clear to a potential reviewer what the format of the work is and what is expected from her or him.

 Specific comments

- Page 4, item 1.1, ‘...and practical approach to positively improve their welfare...’: improve writing, as ‘positively improve is a pleonasm.
- Second last paragraph in item 1.1, page 7, ‘(i) Graphical representation of the experimental...’, formatting is odd. Consider inserting a subtitle for this formatting to make sense. In the same paragraph, prefer four cages instead of 4 cages, for numbers up to ten, use Arabic numbers only when followed by units, otherwise, write numbers in full.
- Last paragraph, item 1.1. Consider rephrasing, as the verb ‘conclude’ is awkward in the introduction of a scientific text.
- 1.2, page 7: Write your hypothesis as a stronger statement, do not use ‘would’, leave statements in present tense.
- Box 1, page 8: Do not use future tense for procedures done during the experiment – this is also the case for other parts of the text, for instance page 12 – please check the full text.
- Box 1, page 8: Instead of mentioning ‘next cohort’, please identify the cohorts.
- End of page 11: This phrase ‘On either side of the pin the experimenter engaged in PH as in P0’ appears twice. Please rewrite for clarity.
- Page 12, 2.6.1.1, first phrase: ‘Behaviour during the tickling sessions were...’ Check grammar.
- Page 13, Table 1, check the word ‘partner’ in the end of the second last line.
- Page 13, objective 2: ‘...quantifying exploration behaviour in two different structures’ instead of  ‘...quantifying exploration behaviour in 2 different structures’. There are many phrases for which this improvement is possible.
- Page 14: Why is ‘Alteration in the experimental procedure’ in the results section?
- Page 16, 3.1.5: ‘...on a proportion of the audio recordings.’ Which proportion? ‘We found strong concordance...’ How strong? Please provide numbers when they are available.
- Page 18, 3.1.6.1: Does tickling*sex interaction mean treatment*sex? If so, keep wording consistent to improve readability; if not, describe with further detail.
- Page 18, 3.1.6.3: Please report in which treatment was the rat that fell sick.
- Page 24: ‘A weak, negative correlation (r = -0.336) was observed between USV production on the final day of treatment and FCM at the end of the treatment phase.’ Please inform p value, as it is relevant to know whether this correlation was significant.
- Page 26: On between sex comparisons: ‘This suggests that except for the P4 treatment, females found the treatments applied here including the control conditions more rewarding than males.’ Does it? Can we also think that females seem more ‘talkative’ throughout? Can it be interpreted otherwise, by recognizing that males only improved their USVs in P4? Please elaborate a little further.
- Page 27: On FCM: perhaps recruiting knowledge regarding the effects of excitation and physical exercise would help discussing the results observed. The discussion on FCM could be more detailed.
- Page 28, 4.3: The single male human playing partner is a very important issue. Consider a paragraph in the discussion section, instead of a simple citation as a limitation of the study, to allow for a more detailed approach. What restrictions did this impose, from the point of view of the rats? What would you expect to be different should the rats be playing with a woman?

Is the work clearly and accurately presented and does it cite the current literature?

Partly

If applicable, is the statistical analysis and its interpretation appropriate?

Yes

Are all the source data underlying the results available to ensure full reproducibility?

Partly

Is the study design appropriate and is the work technically sound?

Yes

Are the conclusions drawn adequately supported by the results?

Yes

Are sufficient details of methods and analysis provided to allow replication by others?

Yes

Reviewer Expertise:

Anima welfare

I confirm that I have read this submission and believe that I have an appropriate level of expertise to confirm that it is of an acceptable scientific standard.

# Reviewer report: Reviewer response for version 1

**DOI:** 10.5256/f1000research.183401.r404436

**Published:** 2025-08-30

**Authors:** Sergio Pellis

**Affiliations:** 1 University of Lethbridge, Lethbridge, Canada

While there is still room for debate as to whether heterospecific tickling truly mimics peer-peer social play, many rats find being tickled by a human rewarding, making such interactions of value for improving the psychological welfare of laboratory housed rats. However, it is important to know exactly which aspects of the tickling procedure rats find rewarding and which may be aversive if this technique is to be most effectively implemented. The present paper provides strong evidence that limiting the number of human-induced ‘pins’ in a tickling session, results in the most rewarding outcomes for rats, at least as measured by their emission of 50kHz calls, especially for females. Interestingly though, some pinning is better than no pinning. Consequently, the modified tickling paradigm proposed by the authors is a refinement to the procedure that could enhance the usefulness of this technique for both welfare and to further understand what it is that rats find pleasurable during playful interactions. The authors note that the proposed modified procedure should be further tested across different strains and ages. Given that a limited number of pins per interaction works best, and there are strains that perform more and strains that perform less pins than do Wistar rats during peer-peer play (Himmler, S. M., Modlińska, K., Stryjek, R., Himmler, B. T., Pisula, W., & Pellis, S. M. (2014). Domestication and diversification: A comparative analysis of the play fighting of the Brown Norway, Sprague-Dawley, and Wistar strains of laboratory rats. *Journal of Comparative Psychology*, 128, 318-327; Himmler, S. M., Lewis, J. M. & Pellis, S. M. (2014). The development of strain typical defensive patterns in the play fighting of laboratory rats. *International Journal of Comparative Psychology*, 27: 385-396), the relative number of pins per tickling session may need to be adjusted. Similarly, when testing young adults, the effect of dominance needs to be considered (Pellis, S. M., Hastings, E., Shimizu, T., Kamitakahara, H., Komorowska, J., Forgie M. L., & Kolb, B. (2006). The effects of orbital frontal cortex damage on the modulation of defensive responses by rats in playful and non-playful social contexts. *Behavioral Neuroscience*, 120, 72-84) as this could again influence the relative number of pins that are optimal in older rats.

 The presentation, execution and analyses of the study is excellent, I have no major concerns. Indeed, the study is so thorough that even the negative results can be believed. I just found one inaccuracy in the narrative that should be addressed, especially if it affects any of the measurements used in this study.

 Page 5, 2 ^nd^ last para: The claim that in social play the actions depicted in Figure 1 ‘can occur in any order’ is not accurate. Pinning (C), chasing (D) and upright (E) are almost always preceded by a nape attack or an attempted nape attack. What is correct is that the type of defensive action following a nape attack can vary and not all such actions need to occur in all play fights (see Pellis & Pellis, 2013 already cited).

Is the work clearly and accurately presented and does it cite the current literature?

Yes

If applicable, is the statistical analysis and its interpretation appropriate?

Yes

Are all the source data underlying the results available to ensure full reproducibility?

Yes

Is the study design appropriate and is the work technically sound?

Yes

Are the conclusions drawn adequately supported by the results?

Yes

Are sufficient details of methods and analysis provided to allow replication by others?

Yes

Reviewer Expertise:

Behavioural neuroscience, animal behaviour

I confirm that I have read this submission and believe that I have an appropriate level of expertise to confirm that it is of an acceptable scientific standard.

## References

[1] : Stage 2 Registered Report: Refinement of tickling protocols to improve positive animal welfare in laboratory rats. *F1000Research* .2025;14: 10.12688/f1000research.166417.1 10.12688/f1000research.166417.1 40963525 PMC12438952

[2] : Domestication and diversification: A comparative analysis of the play fighting of the Brown Norway, Sprague-Dawley, and Wistar laboratory strains of (Rattus norvegicus). *Journal of Comparative Psychology* .2014;128(3) : 10.1037/a0036104 318-327 10.1037/a0036104 24749500

[3] : The Development of Strain Typical Defensive Patterns in the Play Fighting of Laboratory Rats. *International Journal of Comparative Psychology* .2014;27(3) : 10.46867/ijcp.2014.27.03.09 10.46867/ijcp.2014.27.03.09

[4] : The effects of orbital frontal cortex damage on the modulation of defensive responses by rats in playful and nonplayful social contexts. *Behavioral Neuroscience* .2006;120(1) : 10.1037/0735-7044.120.1.72 72-84 10.1037/0735-7044.120.1.72 16492118
